# Proximity-dependent proteomics of the *Chlamydia trachomatis* inclusion membrane reveals functional interactions with endoplasmic reticulum exit sites

**DOI:** 10.1371/journal.ppat.1007698

**Published:** 2019-04-03

**Authors:** Mary S. Dickinson, Lindsey N. Anderson, Bobbie-Jo M. Webb-Robertson, Joshua R. Hansen, Richard D. Smith, Aaron T. Wright, Kevin Hybiske

**Affiliations:** 1 Department of Global Health, Graduate Program in Pathobiology, University of Washington, Seattle, WA, United States of America; 2 Department of Medicine, Division of Allergy and Infectious Diseases, Center for Emerging and Reemerging Infectious Disease (CERID), University of Washington, Seattle, WA, United States of America; 3 Biological Sciences Division, Pacific Northwest National Laboratory, Richland, WA, United States of America; 4 The Gene and Linda Voiland College of Chemical Engineering and Bioengineering, Washington State University, Pullman, WA, United States of America; McMaster University, CANADA

## Abstract

*Chlamydia trachomatis* is the most common cause of bacterial sexually transmitted infection, responsible for millions of infections each year. Despite this high prevalence, the elucidation of the molecular mechanisms of *Chlamydia* pathogenesis has been difficult due to limitations in genetic tools and its intracellular developmental cycle. Within a host epithelial cell, chlamydiae replicate within a vacuole called the inclusion. Many *Chlamydia*–host interactions are thought to be mediated by the Inc family of type III secreted proteins that are anchored in the inclusion membrane, but their array of host targets are largely unknown. To investigate how the inclusion membrane proteome changes over the course of an infected cell, we have adapted the APEX2 system of proximity-dependent biotinylation. APEX2 is capable of specifically labeling proteins within a 20 nm radius in living cells. We transformed *C*. *trachomatis* to express the enzyme APEX2 fused to known inclusion membrane proteins, allowing biotinylation and purification of inclusion-associated proteins. Using quantitative mass spectrometry against APEX2 labeled samples, we identified over 400 proteins associated with the inclusion membrane at early, middle, and late stages of epithelial cell infection. This system was sensitive enough to detect inclusion interacting proteins early in the developmental cycle, at 8 hours post infection, a previously intractable time point. Mass spectrometry analysis revealed a novel, early association between *C*. *trachomatis* inclusions and endoplasmic reticulum exit sites (ERES), functional regions of the ER where COPII-coated vesicles originate. Pharmacological and genetic disruption of ERES function severely restricted early chlamydial growth and the development of infectious progeny. APEX2 is therefore a powerful in situ approach for identifying critical protein interactions on the membranes of pathogen-containing vacuoles. Furthermore, the data derived from proteomic mapping of *Chlamydia* inclusions has illuminated an important functional role for ERES in promoting chlamydial developmental growth.

## Author summary

*Chlamydia* are obligate intracellular bacteria that require the establishment and maintenance of a host vacuole (the inclusion) for their developmental growth. Chlamydiae modify the inclusion membrane through the secretion of type III membrane proteins and recruited host factors. These membrane modifications are predicted to transform the inclusion into a favorable replicating environment through the host protein networks they interact with. Despite the essentiality of the inclusion for *Chlamydia*, only a preliminary understanding exists concerning its membrane protein composition. In particular, the early mechanisms controlling the biogenesis of the inclusion are poorly understood. To develop a deeper understanding of the inclusion membrane interaction proteome, we capitalized on recent advances in *Chlamydia* genetics to express and localize a proximity-dependent biotinylation tag onto type III secreted inclusion membrane proteins. We then applied this system to identify by mass spectrometry the major host and chlamydial proteins recruited to early, mid, and late stage inclusion membranes of *C*. *trachomatis* infected cells. Interestingly, proteomic data showed enrichments of proteins with characterized roles in regulating endoplasmic reticulum exit sites (ERES). Functional testing through protein knockdown and pharmacological inhibition demonstrated that interactions between early *C*. *trachomatis* inclusions and ERES are critical for promoting the developmental growth of *Chlamydia*.

## Introduction

*Chlamydia trachomatis* is an obligate intracellular bacterium that infects mucosal epithelial cells of the endocervix and conjunctiva. It infects millions of people every year and is the etiological agent of ocular trachoma [1,2]. Although *C*. *trachomatis* infections are effectively treated with antibiotics, the majority of infections are asymptomatic and go untreated [3]. The consequences of long term infection can be severe, especially in chronically infected women that are at risk for developing pelvic inflammatory disease, ectopic pregnancy, or infertility as a consequence of infection [4]. Chlamydiae undergo a biphasic developmental cycle, characterized by transitions between infectious elementary bodies (EB) and metabolically active reticulate bodies (RB) [5,6]. During infection, an EB attaches to a host cell and internalizes into a vacuole called the inclusion. Within the inclusion, EB–RB conversion and replication proceed, ultimately followed by asynchronous conversion to EB and exit from the host cell. Chlamydial growth within host cells is critically dependent on recruitment of host proteins to the inclusion membrane early during infection, and on extracting nutrients from the host cell. The mechanisms responsible for these processes are not well understood. The streamlined genome of *Chlamydia* necessitates a dependency on the host cell for many nutrients, yet some of these molecules cannot freely permeate the inclusion membrane and import systems have not been identified [7]. In addition, while much is known about chlamydial manipulation of host signaling, very little is known about the molecular processes necessary for *Chlamydia* to obtain nutrients from the host [6,8].

The inclusion membrane represents the major interface through which chlamydiae manipulate host cell function. In accordance with this, different chlamydial species encode 50–70 type III secreted inclusion membrane (Inc) proteins that are predicted to localize to the inclusion membrane during the chlamydial developmental cycle [9–14]. The Inc family is a signature genetic feature of chlamydiae; however, their lack of sequence similarity with proteins from other bacteria has largely precluded bioinformatic prediction of molecular functions and potential host binding partners. Two major proteomic studies in recent years have greatly advanced our knowledge of candidate host proteins associated with the inclusion membrane and with specific Inc proteins [15,16]. These studies also highlighted molecular interactions that occur between the inclusion membrane and the retromer complex [15,16].

A comprehensive understanding of inclusion membrane modifications, and the host proteins recruited to the inclusion, has not been realized. Even less is known regarding the temporal dynamics of these interactions over the 48-72-hour *C*. *trachomatis* developmental cycle, and the factors that are critical for inclusion biogenesis. Molecular analysis of early inclusions has been particularly elusive due to their small size. Previous proteomic efforts provided major new insight into the inclusion membrane proteome; however, they were unable to characterize protein compositions of early inclusions or identify temporal protein associations. The development of techniques to investigate the inclusion membrane proteome under native conditions, at multiple stages of infection, would accelerate the discovery of *Chlamydia*–host interactions. To this end, we used the APEX2 system of proximity-dependent biotinylation in *C*. *trachomatis*, as a flexible tool for exploring host-pathogen interactions. APEX2 has been tested in *Chlamydia* by microscopy and western blot, and shown to be able to biotinylate proteins on the inclusion membrane when fused to inclusion membrane proteins IncF, IncA, or a truncated IncA [17]. APEX2 is an ascorbate peroxidase that catalyzes a reaction between biotin-phenol and hydrogen peroxide, forming a phenoxyl radical that rapidly forms a covalent bond with a nearby amino acid [18,19]. The labeling radius of APEX2 is less than 20 nm, and the reaction is carried out in living cells for only one minute; this allows highly spatially and temporally resolved biotinylation of proteins in situ. In combination with mass spectrometry, APEX2 has been used to map the mitochondrial matrix, outer membrane, and inner membrane space, as well as the endoplasmic reticulum (ER) membrane and several other subcellular locations of mammalian cells [19–22]. Recently, APEX2 was used to study how protein interactions with G-protein-coupled receptors change after activation, highlighting its experimental utility for studying protein interaction dynamics [23]. We engineered a *C*. *trachomatis* strain that contained an Inc protein fused to APEX2,under the control of an inducible promoter. Using this strain, we expressed Inc-tagged APEX2 on the inclusion membranes of cells infected with *C*. *trachomatis* at three stages of growth. Subsequent quantitative mass spectrometry defined the proteomes of inclusion membrane proximal proteins at early, middle, and late stages of chlamydial development. Analysis of proteomic data for early inclusions showed a significant enrichment of endoplasmic reticulum proteins, in particular factors with established roles in the regulation of ER exit sites (ERES). We detail the recruitment of specific ERES factors to *C*. *trachomatis* inclusions, and we demonstrate that functional ERES are important for chlamydial developmental growth.

## Results

### Development of the APEX2 *in situ* proximity labeling system for *C*. *trachomatis*

We sought to develop the APEX2 system for identifying inclusion interacting proteins, with the primary goal of determining how these interactions evolve during *Chlamydia* infection of host cells. Previous mass spectrometry studies of the inclusion membrane were either done in the absence of infection, or only at a later stage of infection after mechanical manipulations [15,16]. These studies also used techniques that required lysing open the host cells and pulling down Incs or whole inclusions in an in vitro environment, which may have disrupted weaker, more transient protein-protein interactions. With the recent development of a transformation system for *Chlamydia*, a wide range of techniques are now possible [24,25]. Leveraging this advance, we infected cells with a *C*. *trachomatis* L2/434 strain engineered to express flag-tagged Inc-APEX2 fusion proteins, to enable the labeling of inclusion membrane interacting proteins in live cells, at multiple times during infection. The Inc-APEX2 fusion protein localized to inclusion membranes, with APEX2 exposed in the host cell cytosol. The APEX2 system is highly sensitive, has defined the proteomes of cellular compartments refractory to other techniques, and APEX2 enzymatic function was shown to remain intact when tagged to a chlamydial Inc protein [17,18,21].

We transformed *C*. *trachomatis* to express different Inc proteins (IncB, IncA, IncC, InaC, and CT223) tagged to flag-APEX2 and tested protein biotinylation levels by western blot, and flag-APEX localization by immunofluorescence microscopy. Although IncB, IncC, and CT223 are known to localize to microdomains in the inclusion membrane, when overexpressed and fused to APEX2 they often localized around the entire inclusion membrane (S1 Fig). There is no quantitative data in the literature about the frequency of microdomains, so it is unknown if the overexpression is truly causing a difference in localization. IncC-APEX2 was seen in smaller patches of the inclusion membrane in some inclusions and distributed around the inclusion membrane in others (S1 Fig). IncB-APEX2 and CT223-APEX2 did not appear to be exclusively in microdomains; CT223-APEX2 was observed both on the inclusion membrane as well as inside bacteria, while IncB-APEX2 was exclusively observed on the inclusion membrane (S1 Fig). These results may be different than the localization seen by overexpression of flag-tagged Incs, where IncB-flag, CT223-flag, and IncC-flag localized to microdomains; however, without side-by-side comparison of both constructs it is difficult to assess if the localizations were altered by APEX2 [14]. It is possible that APEX2 interfered with endogenous binding partners of these Incs, or expression levels were different than in a previous study [14]. IncA-APEX2 was not secreted very efficiently, but this appears to be an issue with our construct, as IncA-APEX2 has been previously tested [17]. To determine whether overexpression had a negative effect on *Chlamydia* growth we infected HeLa cells with *Chlamydia* strains expressing Inc-APEX2 fusions and induced expression with 1 ng/mL anhydrotetracycline (ATc) at the start of infection, then measured inclusion diameter at 24 hours post infection (hpi). Strains expressing IncA-APEX2 and IncB-APEX2 resulted in the largest inclusions as measured by diameter; IncA-APEX2 was not secreted efficiently so we did not go forward with this strain, but IncB-APEX2 had a clear localization around the inclusion membrane (S1C Fig). We also tested if IncB-APEX2 overexpression affected endogenous Inc localization using immunofluorescence microscopy. By comparing uninduced or IncB-APEX2 expressing *C*. *trachomatis* we did not observe any differences in IncA localization, and microdomains marked with CT223 appeared normal (S2 Fig). There was a slight decrease in inclusion diameter with the IncB-APEX2 *Chlamydia* when grown in the presence of ATc, indicating overexpression had some detrimental effects on inclusion growth (S2C Fig). Since IncB-APEX2 distributed evenly around the inclusion membrane and did not cause expansion of endogenous CT223 positive microdomains, we deemed it the most suitable whole inclusion membrane probe, rather than identifying microdomain-specific proteins [14,26].

To further ensure that the results of IncB-APEX2 were generalizable, we performed preliminary mass spectrometry analysis with the two Incs that gave the highest amount of biotinylation by western blot (IncB and CT223). We determined that IncB-APEX2 identified more host proteins when overexpressed, in concordance with the western blot data, and IncB-APEX2 detected all the proteins that were found by the CT223-APEX2 fusion with the exception of two proteins. This fits with previous uses of APEX2, where the proteomic results were localization-specific, rather than representing binding partners of the protein fused to APEX2. We therefore chose to focus on IncB-APEX2 for the rest of our experiments. In subsequent mass spectrometry experiments IncB-APEX2 identified the two proteins that were identified by CT223-APEX2 in the first trial, confirming that these proteins were not unique to CT223. Furthermore, IncB is constitutively expressed during infection, ensuring that any chaperones necessary for proper secretion will be present at any time points tested [26–28].

**Fig 1:**
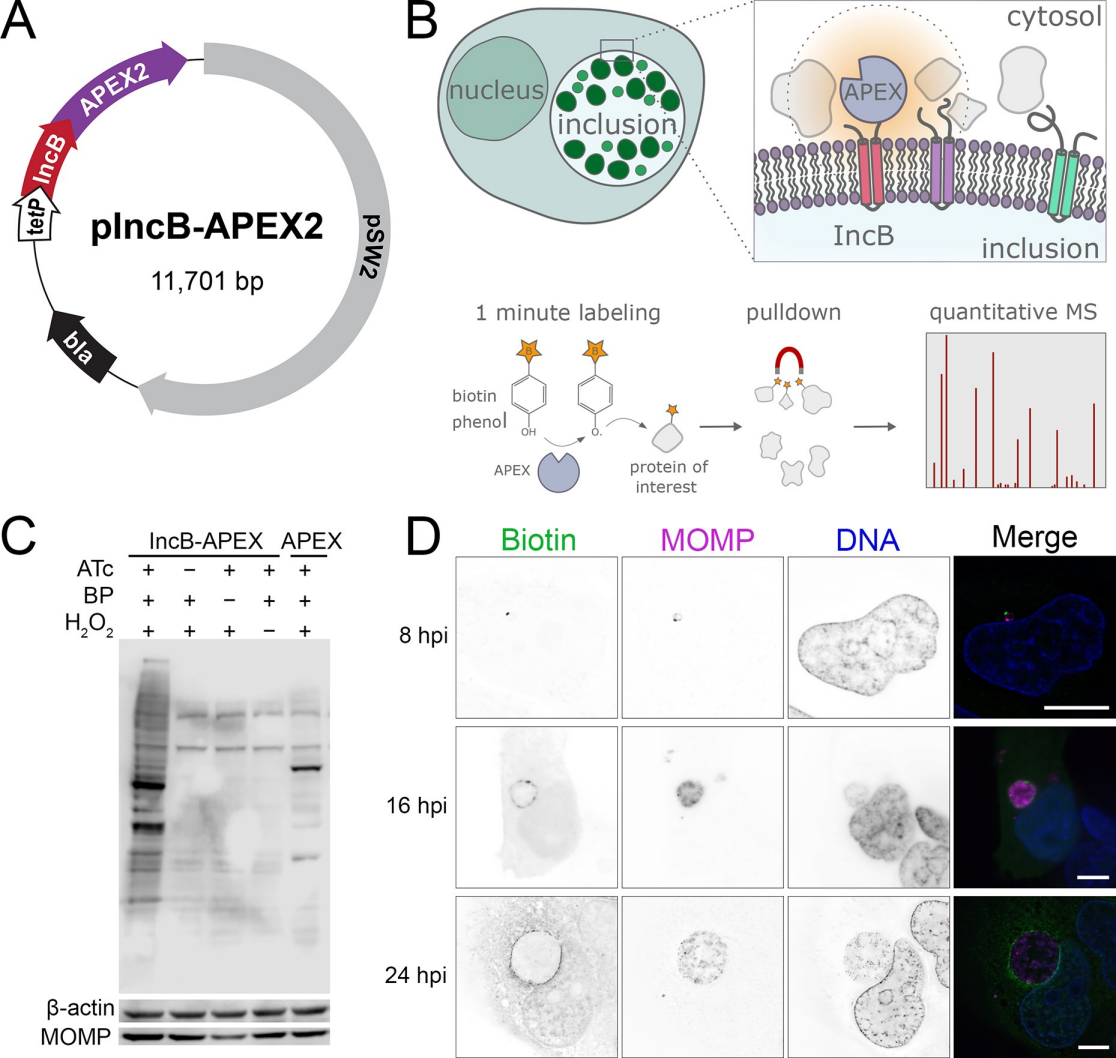
In situ proteomic labeling of the *C. trachomatis* inclusion membrane. (A) Plasmid used to transform *C. trachomatis* L2 and localize APEX2 to the inclusion membrane. IncB-APEX2 fusion expression was under the control of a tetracycline inducible promoter. Flag epitope tag was added between IncA and APEX2. (B) Schematic for APEX2 localization and biotinylation reaction. Cells were infected with *C. trachomatis* expressing IncB-APEX2 for 8, 16, or 24 hours, expression was induced by anhydrotetracycline, and incubation with biotin-phenol and hydrogen peroxide for 1-minute catalyzed biotinylation of proteins within 20 nm of APEX2. Biotinylated proteins were enriched using streptavidin coated agarose resin and relative abundance estimated using mass spectrometry-based proteomics. (C) Western blot analysis of cells infected for 16 hours with *Chlamydia* expressing IncB-APEX2 (first four columns) or untagged APEX2 (last column). Blots were probed with streptavidin-HRP to detect biotinylated proteins. Anti-beta-actin and anti MOMP antibodies were used as human and *Chlamydia* loading controls, respectively. BP, biotin-phenol; ATc, anhydrotetracycline. (D) Immunofluorescence microscopy of cells infected with C. trachomatis IncB-APEX2 and after 1 min inclusion membrane protein labeling at 8, 16, and 24 hpi. Representative images are shown. Biotin labeled proteins were identified by streptavidin-Alexa 488, *Chlamydia* were labeled with an anti-MOMP antibody, DNA labeled with DAPI. Single channel images are displayed in inverted grayscale. Merged panels display all three-color channels. Scale bars = 16 μm.

For mass spectrometry experiments we used *C*. *trachomatis* L2/434 transformed with a tetracycline inducible plasmid encoding IncB-APEX2 (Fig 1A). At specific time points, biotin-phenol and hydrogen peroxide were added to catalyze protein biotinylation, and resulting labeled proteins were pulled down using streptavidin and identified by mass spectrometry (Fig 1B). We confirmed that protein biotinylation required IncB-APEX2 expression, biotin phenol, and hydrogen peroxide (Fig 1C). Furthermore, the profile and depth of labeled proteins in cells infected with the IncB-APEX2 expressing *C*. *trachomatis* strain were distinct from cells infected with a strain expressing an untagged APEX2 that was not secreted (Fig 1C). Staining of infected, labeled cells with a fluorescent streptavidin probe demonstrated that biotinylated proteins were enriched on inclusion membranes at 8, 16 and 24 hours post infection (hpi) (Fig 1D). Biotinylated proteins at 8 hpi manifested as distinct foci which were always outside of the bacterial outer membrane, indicating that IncB-APEX2 is being secreted.

### Identification of inclusion proximal proteins by quantitative mass spectrometry

HeLa cells were infected with IncB-APEX2 *C*. *trachomatis*, with 1 ng/ml anhydrotetracycline added at the start of infection to induce expression [29]. At 8, 16, or 24 hpi, infected cells were incubated with biotin-phenol for 30 min, and protein biotinylation was catalyzed by the addition of hydrogen peroxide for 1 minute. Following reaction quenching, cells were immediately pelleted and frozen for further processing. To control for endogenously biotinylated proteins, control cells were infected and processed in parallel to the biotinylated samples, except no hydrogen peroxide was added so any biotinylated proteins present were not due to the APEX2 reaction. For each infection time point, biotinylated proteins were prepared from six biological replicates and six controls for enrichment and analysis by mass spectrometry.

**Fig 2:**
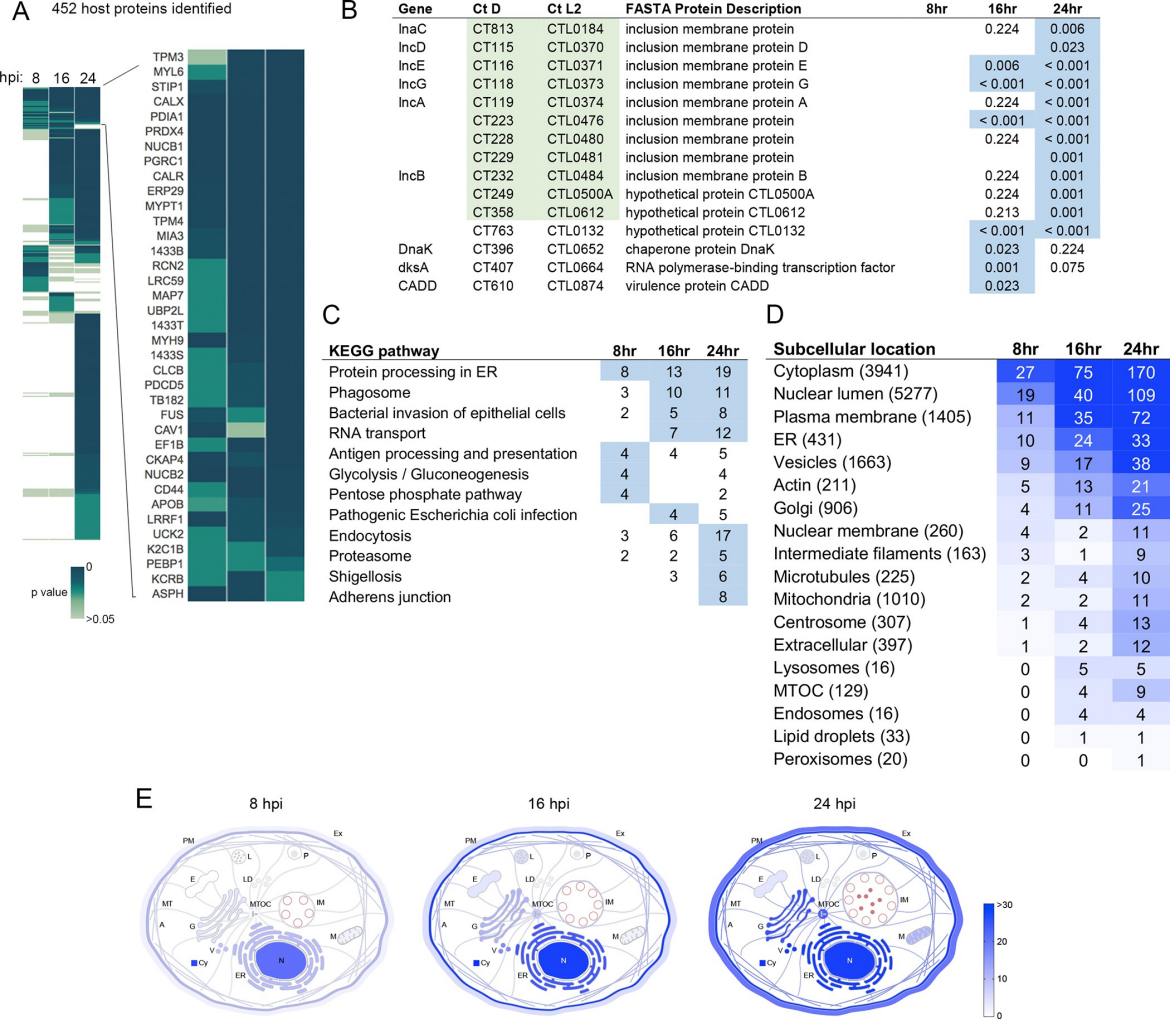
Global analysis of the inclusion membrane interaction proteome throughout the *C. trachomatis* developmental cycle. (A) Heatmap of inclusion membrane interacting proteome identified by APEX2 at 8, 16, and 24 hpi. Data from 6 replicate experiments per time point were averaged and compared against 6 replicate controls at similar times. Colors represent p-values, proteins not detected have no color. Enlarged section of heatmap shows proteins significantly enriched at all time points. Significance determined by t-test or g-test, p < 0.05. (B) *C. trachomatis* ORFs identified on the inclusion membrane, with p values displayed for their presence in 6 replicate samples for each time point. Locus tags highlighted in green represent annotated Inc proteins. Values highlighted in blue represent p values < 0.05. (C) KEGG pathway overrepresentation analysis of inclusion membrane proteome. Overrepresentation determined by hypergeometric algorithm with Benjamini Hochberg method for multiple test correction. Values highlighted in blue represent p values < 0.05. (D) Subcellular location enrichments of APEX2 identified proteins. Color intensity reflects the number of proteins for each location annotation pulled from the Human Protein Atlas database. Numbers in parentheses indicate the total number of reference proteins contained under that annotation in the Human Protein Atlas database. (E) Spatial distribution of inclusion interacting proteins from 8–24 hpi using Human Protein Atlas annotations and manual entry of Inc proteins. Color intensity indicates the number of proteins assigned to location annotations. A, actin filaments; Cy, cytosol; E, endosomes; ER, endoplasmic reticulum; Ex, extracellular/secreted; G, Golgi apparatus; IM, inclusion membrane; L, lysosomes; LD, lipid droplets; M, mitochondria; MT, microtubules; MTOC, microtubule organizing center; N, nucleus; P, peroxisomes; PM, plasma membrane; V, vesicles.

Mass spectrometry of IncB-APEX2-labeled samples identified 452 unique host proteins and 15 chlamydial proteins across the three time points analyzed. The presence of these proteins exhibited notable dynamics over the times tested, for example some only present at a single time point, and others maintained throughout infection (Fig 2A). Among host proteins recruited to *C*. *trachomatis* inclusions, 89 were significantly enriched at 8 hpi, 178 proteins at 16 hpi, and 396 proteins at 24 hpi. There were 37 proteins that maintained enrichment at all three time points (Fig 2A). Of the 15 chlamydial proteins labeled by IncB-APEX2, 11 were annotated as Incs (Fig 2B, Incs highlighted in green). Another, CT610 (CADD), does not have a canonical Inc structure but has been shown to be secreted with an inclusion membrane localization [30]. Of the remaining three *Chlamydia* proteins, two are found in high abundance in the bacteria and thus may be due to labeling of pre-secreted IncB-APEX2. A complete list of proteomic data is contained in S1 Table.

To develop an understanding of the general roles of proteins identified by IncB-APEX2 labeling, and to confirm that our approach labeled pathways and cellular components that *Chlamydia* is known to interact with, we analyzed proteomic data against annotation databases. Pathway overrepresentation analysis was performed using InnateDB with KEGG pathways, and the representation of subcellular locations in the data set was determined using the Human Protein Atlas database [31–33]. KEGG pathway analysis showed that many of the identified proteins were associated with cellular pathways known to play roles during *Chlamydia* infection, including ‘bacterial invasion of epithelial cells’ and endocytosis (Fig 2C). Proteins associated with ER processing were significantly enriched at all three time points. Proteins involved in glycolysis and the pentose phosphate pathway were significantly enriched at 8 hpi. Analysis of subcellular localization annotations for IncB-APEX2 labeled proteins revealed a general spatial context consistent with the known perinuclear residence of the inclusion (Fig 2D and 2E) [8]. Ontology analysis indicated that early in *C*. *trachomatis* infection, at 8 hpi, inclusions acquired proteins normally localized to the cytosol, nucleus, plasma membrane, ER, and vesicles. Growth and maturation of the inclusion, at 16 and 24 hpi, was accompanied by a sustained enrichment of proteins associated with early inclusions, as well as an emergence of interactions with cytoskeleton associated proteins: actin, microtubules, centrosomes, the MTOC, and intermediate filaments. Although there are limitations to the accuracy of annotations in KEGG or localization databases, the pathway overrepresentation analysis highlights expected enrichments in the IncB-APEX2 dataset, as well as potential new associations that could be relevant to *Chlamydia* growth.

**Fig 3:**
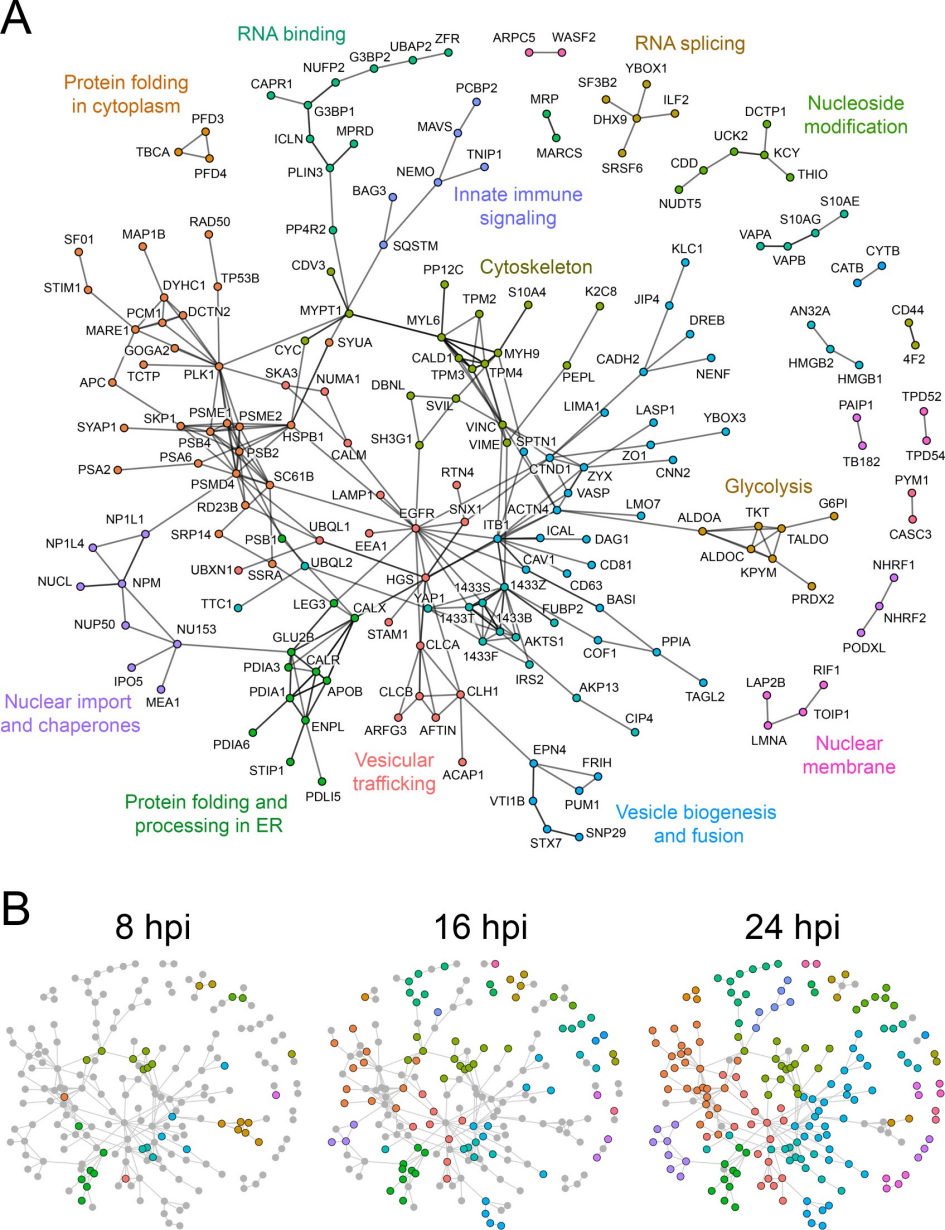
Network analysis of *C. trachomatis* inclusion membrane interacting proteins. (A) Interactions between host proteins identified by APEX2 proteomic labeling were obtained from StringDB [89], and visualization map was generated using R with the tidygraph package [86]. Colors represent more interconnected protein communities within the network. The interaction map in A represents the global extent of inclusion membrane interactions identified over the chlamydial developmental cycle. Edges between protein nodes represent interactions that were curated in StringDB either from published experiments or other databases. Proteins which do not have characterized interaction partners (i.e. single nodes) and translation related proteins were omitted for clarity. (B) Temporal dynamics of inclusion membrane protein interaction networks over the developmental cycle. Protein networks present in 8, 16, and 24 hpi proteomic data sets are represented by colored nodes. Gray nodes depict proteins present in the global interaction map (A) but absent from a specific stage of infection.

We used the STRING database to identify known molecular interactions between identified proteins, and R to plot all proteins with at least one interacting partner (Fig 3A). This allowed analysis of the potential recruitment of multiprotein interaction complexes to the *C*. *trachomatis* inclusion. Highly interconnected protein communities were identified using the Louvain method of community detection. From this analysis, major protein networks emerged, encompassing innate immune signaling, the nuclear membrane, vesicular traffic, the cytoskeleton, nucleoside metabolism, and ER chaperones. Temporal analysis of these interactions revealed a dramatic recruitment of glycolytic proteins to early inclusions, for example aldolases, transketolase, pyruvate kinase, glucose-6-phosphate isomerase, and peroxiredoxin (Fig 3B). Inclusion maturation was marked by a significant expansion of protein networks related to the MTOC and centrosome, innate immune signaling, proteasome regulation, and clathrin assembly (Fig 3B). Network analysis also allowed identification of host factors that may potentially interact with known host–Inc interactions, for example VAPA associates with IncV [34] and the STRING database has evidence that VAPA associates with S10AG and S10AE.

### Functional validation of targets recruited to early inclusions

**Fig 4:**
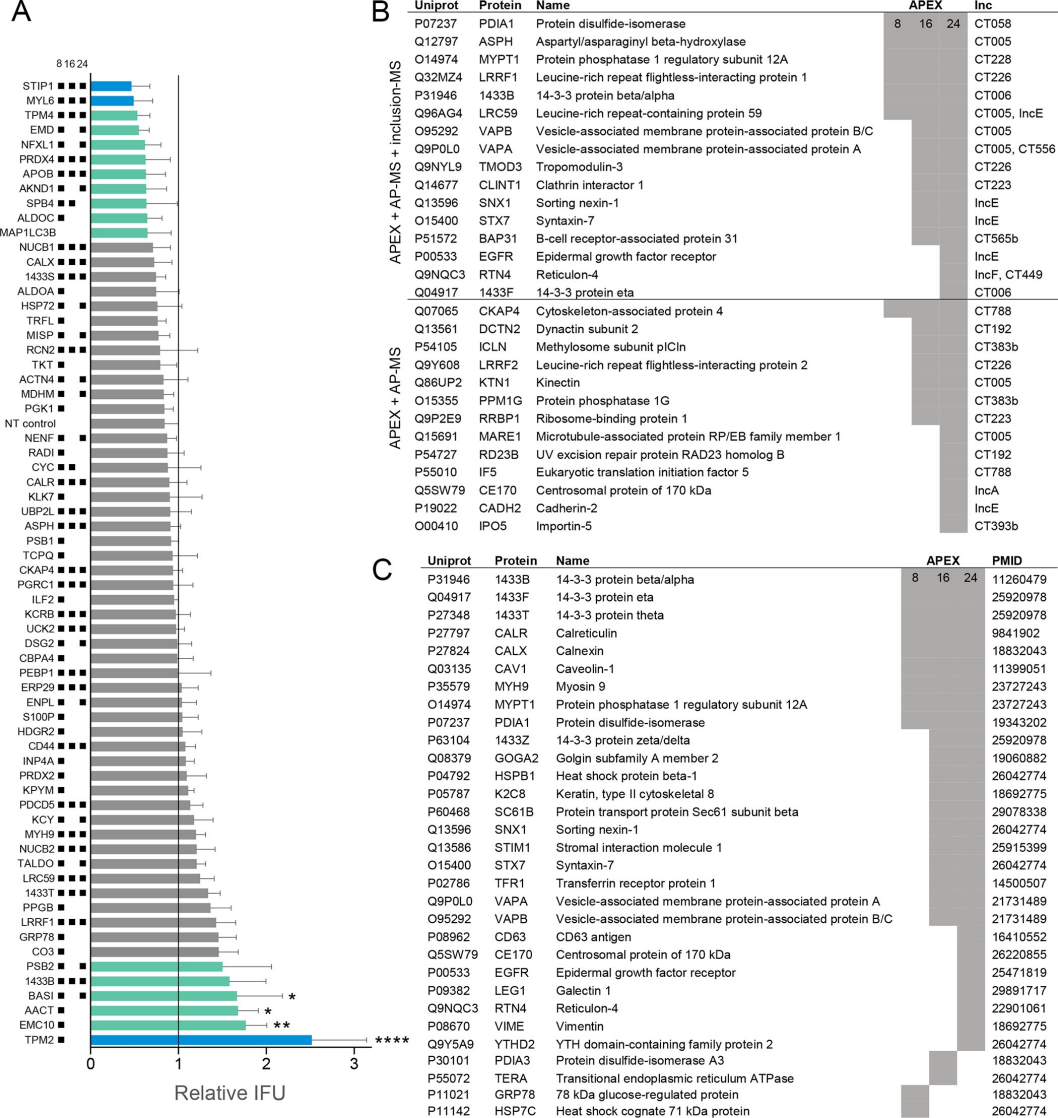
RNAi validation of inclusion interacting proteins and comparison to previous data sets. (A) IFU determination following RNAi depletion of 64 proteins identified from early (8 hpi) inclusions. Cells were transfected with siRNA corresponding to targets shown on the y-axis, infected with *C. trachomatis* L2, and harvested for IFU determination at 48 hpi. Bars mean(SD); n = 3; IFU shown relative to mean IFU of plate. Black squares next to RNAi targets (y-axis) indicate which time points the protein was enriched in APEX2 mass spectrometry data. Teal bars correspond to at least 1.5-fold increase/decrease compared to the mean, blue bars are over 2 fold increase/decrease compared to the mean. Significance was determined by one-way ANOVA with Dunnett’s multiple comparisons test, comparing to mean infectivity of plate; *, p < 0.05; **, p < 0.01; ****, p < 0.0001. (B) Summary of all proteins shared between APEX2 data and two previous inclusion mass spectrometry data sets [15,16]. Lower table describes all proteins that overlap between APEX2 and AP-MS Inc-specific interactions [15]. Shaded boxes represent significant enrichment found by APEX2. (C) Summary of APEX2 identified proteins with reported microscopy-based associations with *Chlamydia* inclusions. PMID entries refer to the publications used to provide this evidence.

Because *C*. *trachomatis* inclusions at 8 hpi are so small, validation of host protein recruitments by fluorescence microscopy is difficult. We reasoned that proteins associated with early inclusion membranes might play important roles in mediating the biogenesis of the inclusion. To validate the functional participation of inclusion-associated proteins towards *Chlamydia* developmental growth, we performed RNA interference (RNAi) on 64 of the 89 proteins present in the 8 hpi interaction dataset. HeLa cells were grown in a 96 well plate format, transfected with siRNA oligonucleotides for 48 h, and infected with *C*. *trachomatis* L2 to determine the impact of protein depletion on bacteria growth. At 48 hpi, EB were harvested from siRNA treated cells and analyzed for inclusion forming units (IFU) on fresh cells. Knockdown of 16 proteins (25%) resulted in a >1.5-fold change in infectious progeny formation, as compared to the mean IFU of the plate and a nontargeting siRNA control (Fig 4A). A change of 1.5-fold was highlighted due to use in a previous siRNA screen [35]. As a positive control, depletion of MAP1LC3B resulted in decreased *C*. *trachomatis* IFU, consistent with reported findings [35]. Overall, 10 protein knockdowns resulted in a >1.5-fold decrease in IFU (Fig 4A top), and 6 protein knockdowns led to a >1.5 fold increase in IFU (Fig 4A bottom). We verified knockdown by qRT-PCR for the targets with at least 1.5-fold difference in IFU (S3 Fig). The knockdown efficiency was over 75% for 11 of the 16 targets, so some of the knockdowns in this screen may be underestimating the effect of depletion on IFU due to low knockdown efficiency. Proteins whose depletion resulted in decreased IFU were frequently targets identified by IncB-APEX2 at multiple stages of *Chlamydia* development (Fig 4A). In contrast, many of the proteins whose knockdown led to increased IFU production were more transiently recruited to inclusions, with enrichments primarily detected at early inclusions (Fig 4A). Collectively, the RNAi data show IncB-APEX2 identified proteins at the inclusion membrane that functionally impacted chlamydial growth.

Our mass spectrometry data provided an opportunity to synthesize it with those generated by previous studies, to develop a more comprehensive understanding of the protein networks recruited to the inclusion membrane. First, we compared our IncB-APEX2 data set to two previous mass spectrometry experiments that conducted analysis on purified inclusions (labeled as ‘inclusion-MS’ in Fig 4B) [16], and affinity purified Incs expressed in 293 cells (labeled as ‘AP-MS’ in Fig 4B) [15]. Furthermore, proteins were manually cross-referenced against the literature to identify evidence-based reports of proteins recruited to inclusions or inclusion membrane proteins (labeled with PMID in Fig 4C). Overall, 16 proteins were identified by all three MS approaches to interact with inclusions, and the present study now provides temporal data for when these interactions occur during host cell infection (Fig 4B, upper half). Ten of the 16 proteins were previously reported to be recruited to inclusion membranes by immunofluorescence microscopy (Fig 4C). The remaining 6 proteins identified by all 3 MS approaches–ASPH, LRRF1, LRC59, TMOD3, CLINT1, and BAP31–represent priority targets for further study in the context of *Chlamydia* infection. IncB-APEX2 labeling contained 60 additional proteins that were identified on 24 hpi inclusions by inclusion-MS (S1 Table) [16]. Thirteen additional proteins from IncB-APEX2 data correlated with Inc specific data obtained from AP-MS, thus adding in situ context to previously described molecular interactions (Fig 4B, lower half) [15]. Finally, IncB-APEX2 identified 31 proteins previously shown by microscopy to be in close proximity to inclusions during infection (Fig 4C). This substantiates the efficacy of the APEX2 approach and additionally provides important new temporal information for how these host targets are dynamically recruited to the inclusion membrane by *Chlamydia*.

### Recruitment of ERES proteins Sec16 and Sec31 to *C*. *trachomatis* inclusions

IncB-APEX2 proteomic analysis revealed a significant enrichment of ER associated proteins near inclusion membranes throughout infection. These findings are consistent with previous reports of membrane contacts between inclusions and the ER [36,37]. IncB-APEX2 data showed that ER protein associations were present near early inclusions, at 8 hpi, and additionally highlighted an enrichment of markers for ER exit sites (ERES). ERES are specialized subdomains of the ER from which COPII coated vesicles bud and traffic to the ER-Golgi intermediate compartment (ERGIC) [38]. Sec16, TANGO1, TFG, and peflin, were identified on inclusion membranes by IncB-APEX2, with TANGO1 present at all three time points (S1 Table). Sec16 is thought to act as a critical ERES scaffold in mammalian cells and is capable of stabilizing COPII subunits at ERES membrane regions [39]. TANGO1 recruits cargo to ERES through interactions with Sec16 and cTAGE5 [40,41], and TFG promotes COPII uncoating of vesicles prior to fusion with the ERGIC [42]. In support of an intimate association between ERES and inclusions, five members of the p24 family, proteins which regulate ERES organization and are packaged into COPII vesicles [43], were identified by inclusion-MS and AP-MS [15,16].

**Fig 5:**
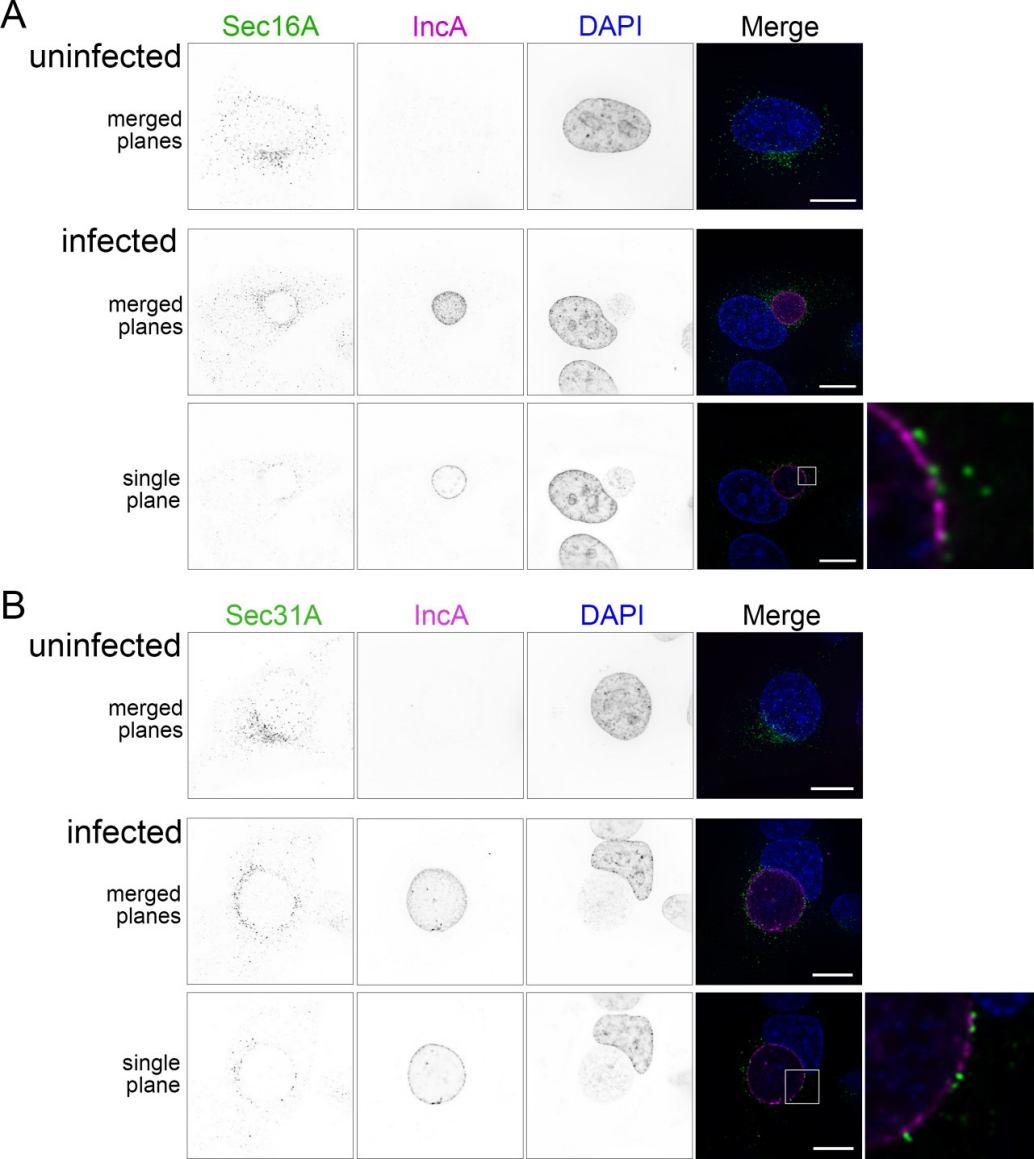
ERES proteins Sec16A and Sec31A are recruited to the *C. trachomatis* inclusion. (A) Immunofluorescence microscopy of cells showing cellular distribution of Sec16A (anti-Sec16A, first column, green in merge), *Chlamydia* IncA (anti-IncA, second column, magenta in merge), and DNA (DAPI, third column, blue in merge). (B) Distribution of the COPII outer coat protein Sec31A (anti-Sec31A, first column, green in merge), IncA, and nuclei. Single channel images are displayed in inverted grayscale. Merged panels display all three-color channels. Protein distribution in uninfected HeLa cells are shown in the top row. Deconvolved images from cells infected with *C. trachomatis* L2 at 24 hpi are shown as a summation of z-series images (merged planes) or a single xy plane. Enlargements (far right) represent the regions marked with white boxes. Scale bars = 16 μm.

To validate these findings, we used immunofluorescence microscopy to investigate the spatial relationship between the ERES marker Sec16 and the inclusion membrane. In uninfected cells, Sec16 was distributed in a normal pattern throughout the cytoplasm in small punctae, with a dense cluster of Sec16 in the perinuclear region (Fig 5A). In *C*. *trachomatis* infected cells, Sec16 redistributed in a punctate pattern around the inclusion at 24 hpi (Fig 5A). We quantified the redistribution of the dense perinuclear cluster of Sec16 around the inclusion membrane using CellProfiler to identify the dense areas of Sec16 and determine how far around the inclusion they are distributed (S4A Fig) [44]. The mean fraction of the inclusion perimeter surrounded by clustered Sec16 was 0.577 (SD = 0.210). About 50% of inclusions had at least 70% or more of the perimeter surrounded by clustered Sec16. Several Sec16 punctae closely abutted inclusion membranes defined by IncA (Fig 5A, inset). Sec16 was also recruited to inclusions at 14 hpi (S5 Fig). We further examined the ectopic distribution of Sec16 in live cells using HeLa cells transfected with Sec16-GFP and infected with a *C*. *trachomatis* strain expressing mCherry and observed a similar association of Sec16 with inclusions (S6 Fig). Next, we tested whether the COPII coat protein Sec31 associated with inclusion membranes in a similar manner, and as would be expected for functional ERES. Like Sec16, Sec31 was redistributed around inclusion membranes, with some Sec31 foci appearing to overlap with the inclusion membrane (Fig 5B, S5 Fig). We quantified Sec31 clusters in the same way as Sec16, and the mean fraction of the perimeter surrounded was 0.66 (SD = 0.247), with 56% of inclusions having at least 70% of the perimeter surrounded by Sec31A clusters (S4B Fig). Together, these data demonstrate that structural and regulatory ERES proteins are recruited to inclusion membranes. It is unclear if these ERES proteins are on the ER, on COPII vesicles, or on the inclusion membrane. The localizations of ERES, ERGIC, and cis Golgi network, are closely related in mammalian cells. Secretory cargo in COPII-coated vesicles leave the ER at ERES, quickly fuse with the ERGIC, and then are packaged into COPI-coated vesicles for transport to the Golgi. To check if the Golgi was necessary for the distribution of ERES to *Chlamydia* inclusions, we resolved the localization of the Golgi and ERES in infected cells with or without Brefeldin A (BFA) treatment (S7 Fig). In untreated cells, the general localization of Sec31 and Golgi were similar, although the individual components did not colocalize. After BFA treatment, the Golgi was dispersed, whereas Sec31 remained distributed in close proximity to inclusions.

### Chemical inhibition of ERES cargo loading restricts *C*. *trachomatis* developmental growth

**Fig 6:**
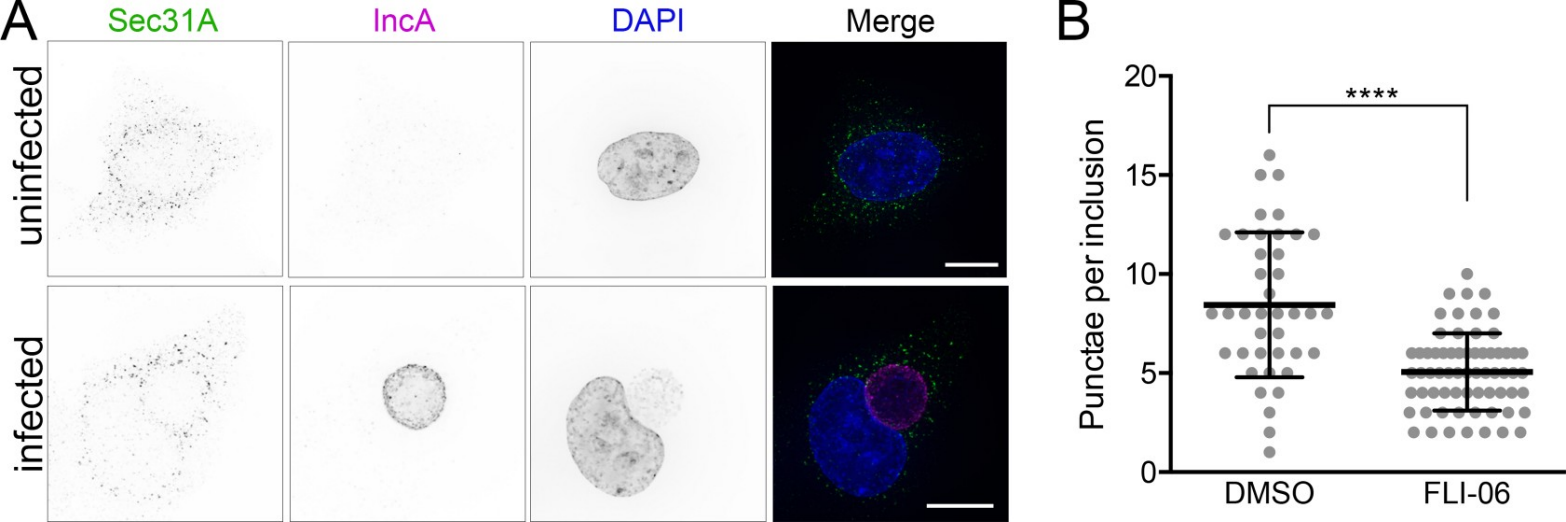
Inhibition of ERES cargo loading abrogates ERES recruitment to the inclusion. (A) Treatment of *C. trachomatis* infected cells with FLI-06 disrupted the recruitment of COPII coat protein Sec31A to inclusion membranes. Immunofluorescence microscopy of cells showing cellular distribution of Sec31A (anti-Sec31A, first column, green in merges), inclusion membrane protein IncA (anti-IncA, second column, magenta in merges), and DNA (DAPI, third column, blue in merges). Representative deconvolved merged z-series images of uninfected cells are shown in upper panels, and cells infected with *C. trachomatis* L2 at 24 hpi are shown in lower panels. 10 μM FLI-06 treatment for 4 h, from 20–24 hpi, resulted in Sec31A distribution similar to that of uninfected cells, and away from inclusion membranes. Scale bars = 16 μm. (B) Quantification of Sec31 in cells infected and treated with FLI-06 as described in A. Sec31 punctae that were touching or overlapping with IncA in untreated or FLI-06 treated cells were counted using Volocity to assess the number of overlapping spots per inclusion. At least 20 inclusions were analyzed per condition, for two independent experiments. Each dot represents an inclusion, lines represent mean (SD). Significance determined by unpaired t-test with Welch’s correction, ****, p < 0.0001.

We next tested if functional ERES were required for *Chlamydia* infection, by using a specific inhibitor of ERES export, FLI-06 [45,46]. FLI-06 is a cell permeable, reversible inhibitor of ERES cargo loading and the early secretory pathway. The molecular target of FLI-06 target is unknown; however, the compound has been shown to prevent cargo recruitment to ERES. Any cargo already recruited is secreted, but no new cargo can be loaded into exit sites. First, we investigated how the localization of Sec16 and Sec31 were affected by incubation of *C*. *trachomatis* infected cells with FLI-06. Treatment of infected cells for 4 h with FLI-06, from 20–24 hpi, abrogated the recruitment of both of these ERES proteins to inclusions, resulting in diffuse localization similar to that seen in uninfected cells treated with FLI-06 (Fig 6A, S8 Fig). Similar effects were observed with live cells expressing Sec16-GFP (S5 Fig). The number of Sec31 punctae that overlapped with IncA were counted and confirmed that treatment with FLI-06 resulted in significantly reduced COPII coat protein recruitment to inclusion membranes (Fig 6B). These data indicate that Sec16 and Sec31 recruitment to inclusions are a functional consequence of COPII vesicle formation, and not a byproduct of their proximity to ER membranes.

**Fig 7:**
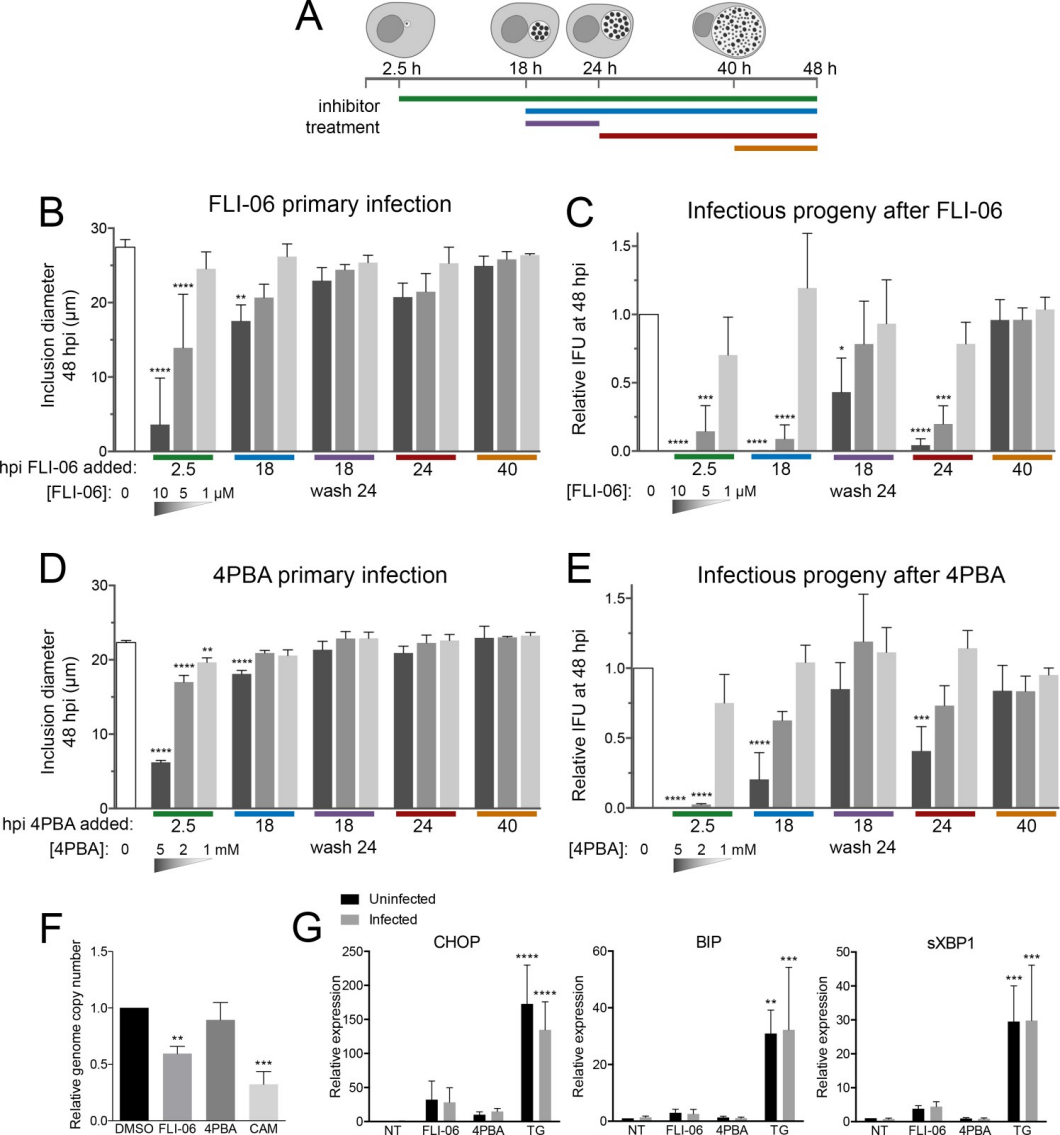
Inhibition of ERES cargo loading or specificity reduces *C. trachomatis* developmental growth. (A) Experimental design used to test the impact of ERES disruption on *Chlamydia* developmental growth. Colored bars mark the times when FLI-06 or 4PBA was applied to infected cells. All cells were harvested for IFU determination at 48 hpi. The effects of ERES inhibition were determined for (B,D) primary infection, through measuring the diameters of inclusions at 48 hpi, or (C,E) IFU production at 48 hpi. Bars denote the mean (n = 3; SD); white bars correspond to untreated controls; gray bars are inhibitor treated in decreasing concentration, 10 μM, 5 μM, or 1 μM FLI-06 and 5 mM, 2 mM, 1 mM 4PBA. Colored bars on x-axis correspond to the inhibitor application key in A. Significance determined by one-way ANOVA with Dunnett’s multiple comparisons test, comparing to untreated control. *, p < 0.05; **, p < 0.01; ***, p < 0.001; ****, p < 0.0001. (F) Infected cells were treated from 24–48 hpi with 10 μM FLI-06, 5 mM 4PBA, or 0.5 μg/mL chloramphenicol (CAM). Genomic DNA was extracted, and genome copy number was determined by using quantitative PCR for the GroEL2 gene. Significance was determined by one-way ANOVA with Dunnett’s multiple comparisons test, comparing to control. **, p < 0.01; ***, p < 0.001. (G) Relative expression of genes upregulated during ER stress in infected or mock infected cells after treatment with 10 μM FLI-06, 5 mM 4PBA, or 1 μM thapsigargin from 20–24 hpi. Gray bars are infected, black are uninfected. Expression was determined using quantitative PCR. Significance was determined using a two-way ANOVA with Dunnett’s multiple comparisons test. There was no significant difference between infected and uninfected cells, thapsigargin treatment was significantly different from the control. Uninfected treatments were compared to uninfected control, infected treatments compared to infected control. **, p < 0.01; ***, p < 0.001; ****, p < 0.0001.

To explore this outcome further, we tested the effect of FLI-06 on chlamydial developmental growth. *C*. *trachomatis* infected cells were treated with three concentrations of FLI-06 at distinct stages of infection: 2.5, 18, 24, and 40 hpi (Fig 7A). Following treatment, the effects of FLI-06 on primary infection and infectious progeny formation were determined by measuring inclusion diameter and IFU, respectively, at 48 hpi for all treatment groups (Fig 7B and 7C). We measured a significant, dose-dependent reduction in inclusion diameter for infected cells treated starting at 2.5 or 18 hpi (Fig 7B, S9 Fig). Inhibitory effects were most pronounced with 10 μM of FLI-06, a concentration previously shown to block the recruitment of VSVG cargo to ERES [45,46]. The effects of FLI-06 were reversible, as treatment of infected cells at 18 hpi followed by washout at 24 hpi resulted in a recovery of inclusion diameter at 48 hpi (Fig 7B). For cells treated with 10 μM FLI-06 at 2.5 hpi, fully formed inclusions at 48 hpi were rarely observed, indicating that early stages of *Chlamydia* and inclusion growth are reliant on COPII vesicle production from ERES. Two to 10-fold lower concentrations of FLI-06 resulted in dose-dependent phenotypes, thus strengthening the support that the effects of FLI-06 were biological.

The impact of FLI-06 on *C*. *trachomatis* IFU formation was more pronounced. Treatment of infected cells with 10 μM FLI-06 at 2.5 hpi resulted in no infectious progeny production, and a 99.8% or 95.6% reduction in *Chlamydia* IFU was observed in cells treated with 10 μM FLI-06 at 18 or 24 hpi, respectively (Fig 7C). Similar to the effects on primary infection, the impact of FLI-06 on *Chlamydia* growth was dose dependent. Cells pulsed with FLI-06 from 18–24 hpi partially recovered from the treatment, though IFU was reduced by 57.0% compared to untreated cells; this indicates that replication was reduced even during a 6-hour treatment. No effect on IFU production occurred when FLI-06 was applied to cells at 40 hpi, indicating that FLI-06 is not directly toxic to *Chlamydia* and that the effects of ERES on chlamydial inclusions were not on EB viability or infectivity. The potent effects of ERES disruption on inclusions at 18 hpi, when inclusions contain mostly RB, were consistent with an inhibitory effect that most heavily impacts RB growth. The application of FLI-06 at 18 hpi prevented the population of bacteria at that stage from converting into infectious EB by 48 h. Taken together, the data demonstrate that early *C*. *trachomatis* inclusions acquire vital cellular components from ERES-derived COPII vesicles for their complete developmental growth.

### Chemical inhibition of ERES cargo sorting reduces *Chlamydia* growth

Since FLI-06 blocks secretion from ERES, it has severe effects on downstream steps of the secretory pathway and disrupts the Golgi [45]. *Chlamydia* is known to acquire sphingomyelin and cholesterol from the trans Golgi network. To test whether Golgi disruption contributed to the effects of FLI-06 on chlamydial growth, we used a chemical inhibitor with a different mechanism from FLI-06. Sodium phenylbutyrate (4PBA) has been used for many years to relieve ER stress by preventing accumulation of misfolded proteins within the ER. Recently it was shown that the secretion of misfolded proteins during 4PBA treatment happens through decreased selectivity of COPII cargo [46]. 4PBA binds the p24 family of proteins, which provide cargo specificity for COPII vesicles, concentrating secreted proteins into vesicles while excluding ER resident proteins [47]. COPII vesicles produced in cells treated with 4PBA show dramatically increased levels of ER resident proteins, along with decreased levels of normal COPII cargo [47].

We repeated the same experiments done with FLI-06 using 4PBA instead and saw similar results on both inclusion diameter and IFU (Fig 7D and 7E). When 4PBA was added very early during infection, inclusions were significantly smaller (Fig 7D) and almost no viable EB were produced (Fig 7E). Consistent with 4PBA having a less severe effect on ERES compared to FLI-06, IFU of cells treated at 18 or 24 hpi with the highest dose of 4PBA was 79.5% or 59.25% reduced, respectively, much less severe than cells treated with FLI-06 at the same times. IFU was not affected for cells treated with 4PBA from 18–24 hpi then washed off, or cells treated starting at 40 hpi, supporting that 4PBA is not directly toxic to *Chlamydia*. Since 4PBA allows protein secretion and still affects *Chlamydia* growth, this indicates that the effects of FLI-06 were unlikely to be a nonspecific effect of blocking protein secretion of the ER or Golgi, and COPII cargo regulation is important for the *Chlamydia* developmental cycle.

When FLI-06 or 4PBA were added early during infection, the inclusion diameter and IFU correlated well, with both being significantly lower than the control. When added at 24 hpi, however, both inhibitors did not significantly affect inclusion diameter, yet IFU for FLI-06 was reduced by 95.6% and for 4PBA was reduced by 59.25% (Fig 7C and 7E). We tested whether the reduction in IFU was due to fewer bacteria present, or if it resulted from disrupted RB-EB conversion. We infected cells with *C*. *trachomatis*; at 24 hpi we added 10 μM FLI-06, 5 mM 4PBA, or 0.5 μg/mL chloramphenicol, then incubated until 48 hpi, extracted genomic DNA and used qPCR to measure *Chlamydia* genome copy number (Fig 7F). As expected, chloramphenicol inhibited *Chlamydia* replication and caused a 67.7% reduction in the number of bacterial genomes present, compared to the control. FLI-06 caused a 40.4% reduction in genome copy number, and 4PBA genome copy number was not significantly different from the control. This indicates that adding FLI-06 at 24 hpi caused some reduction in bacterial replication, but the drastic reduction in IFU must at least in part be due to reduced bacterial infectivity after treatment. 4PBA had a less severe effect on IFU, and the genome copy number supports that replication was not significantly reduced when added at 24 hpi, but there is a reduction in bacterial infectivity.

Given other known effects of 4PBA, it is possible that its effect on *Chlamydia* growth was due to decreased ER stress rather than dysregulation of COPII cargo. Because FLI-06 blocks secretion from the ER, it is also possible that ER stress was induced during FLI-06 treatment and resulted in a detrimental effect on *Chlamydia* growth. We checked if ER stress is induced during *C*. *trachomatis* infection under normal conditions, or after 4PBA or FLI-06 treatment. We compared ER stress in uninfected or *Chlamydia*-infected HeLa cells at 24 hpi after a 4 hour treatment with FLI-06, 4PBA, or thapsigargin, a well described inducer of ER stress [48]. ER stress was assessed by mRNA expression of CHOP, BIP, and spliced XPB1. No significant differences were found between ER stress levels in infected cells and uninfected cells for all conditions tested (Fig 7G). Although CHOP, BIP, and sXBP1 expression were increased in FLI-06 treated cells, this increase was not statistically significant. Thapsigargin was able to induce much higher expression of CHOP, BIP, and sXBP1. Overall, these data indicate that it is unlikely that 4PBA or FLI-06 are acting by altering the levels of ER stress in infected cells.

### *Chlamydia* interactions with ERES are distinct from other interactions with the ER and Golgi

**Fig 8:**
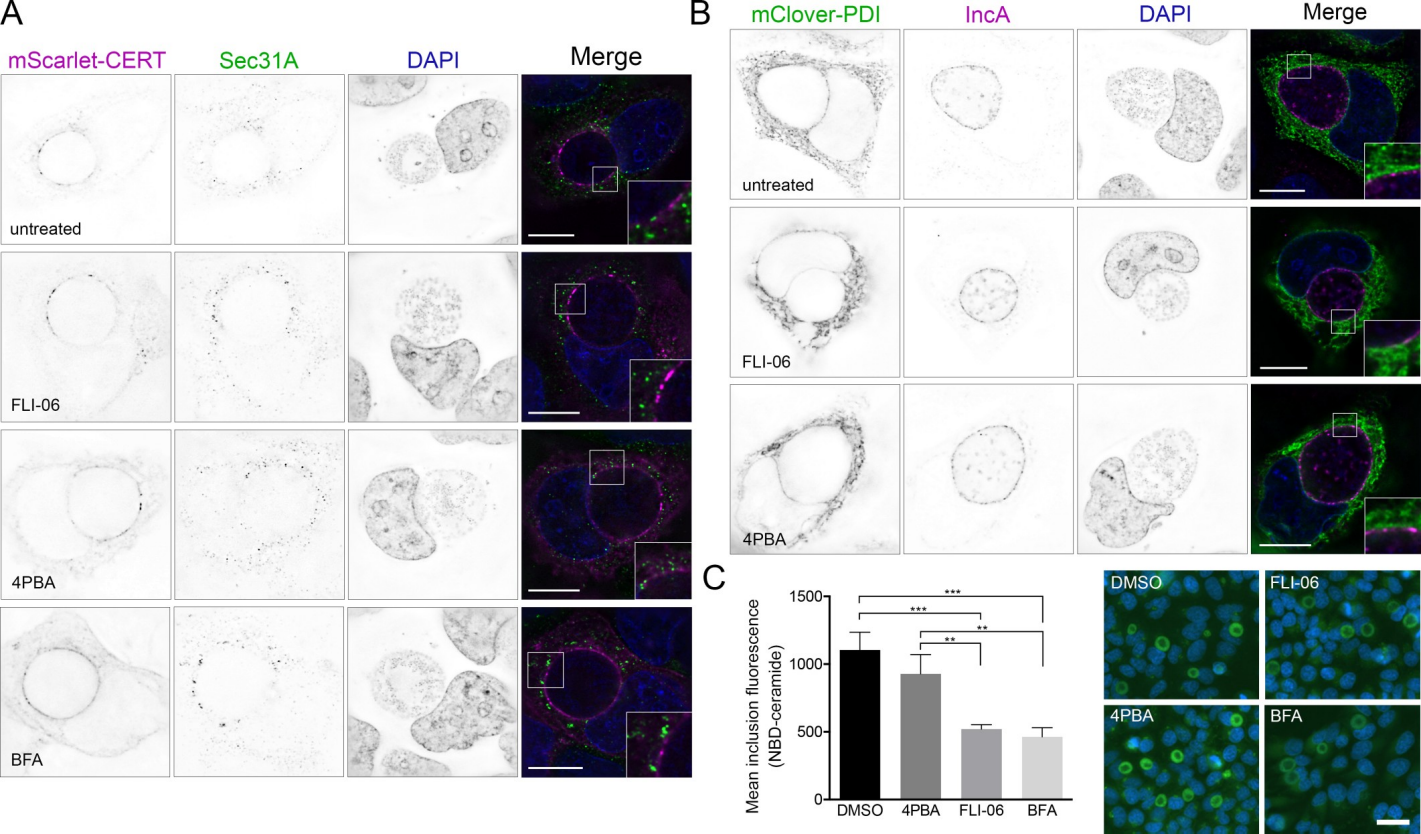
Membrane contact sites and ceramide uptake are maintained during inhibition of ERES. Cells were infected with *C. trachomatis*, then transfected with a plasmid expressing pmScarlet-CERT (A, purple in merge) or pmClover3-PDI (B, green in merge). At 20 hpi, DMSO, 10 μM FLI-06, 5 mM 4PBA, or 3 μg/mL BFA were added to cells. At 24 hpi, cells were fixed and stained with anti-Sec31A (A, green in merge) or anti-IncA (B, purple in merge). DNA was stained with DAPI (blue in merge). Images are single planes from deconvolved z-series. Scale = 16 μm. (B) Inset shows regions along the inclusion membrane where PDI was closely apposed or overlapping IncA. (C) Average inclusion fluorescence after incubation with NBD-C6-Ceramide. Representative images shown on right, NBD-ceramide in green, DAPI in blue. Significance determined by one-way ANOVA with Tukey’s multiple comparison test. Any comparisons not shown were not significant. **, p < 0.01; ***, p < 0.001.

*C*. *trachomatis* inclusions form membrane contact sites with the ER, and whether ERES are recruited by the same mechanism is unclear [36,37]. Previously, the lipid transfer protein CERT was shown to be a key component of ER membrane contact sites with inclusions [36,49]; however, CERT did not colocalize with ERES markers (Fig 8A), indicating that ERES are likely an additional type of ER–inclusion interaction. Since FLI-06 and 4PBA act on the ER, it is possible they could indirectly affect *Chlamydia* growth by reducing contacts between the inclusion membrane and ER. To test this, we overexpressed fluorescent protein tagged CERT or the ER resident protein PDI and saw no difference in their recruitment to inclusion membranes after treatment with FLI-06 or 4PBA (Fig 8A and 8B). We quantified the overlap between IncA and CERT or PDI using Manders’ coefficients and there were no significant reductions in CERT or PDI overlap with IncA following treatment with 4PBA or FLI-06, compared to DMSO (S10 Fig) [50]. BFA was included as a control to ensure any effects were due to the ER rather than post-ER trafficking steps.

CERT recruitment to the inclusion membrane is thought to facilitate non-vesicular ceramide trafficking, whereas the Golgi provides a vesicular route for ceramide uptake into the inclusion. To ensure that these inhibitors did not act through downstream effects on the Golgi, we evaluated Golgi morphology in infected cells treated with 4PBA, FLI-06, or BFA from 20–24 hpi. As expected, the Golgi marker GM130 was distributed around the inclusion in control cells and those treated with 4PBA, while cells treated with BFA and FLI-06 had faint, diffuse GM130 staining (S11 Fig). Although the Golgi was not disrupted by 4PBA, it is possible that there is still an effect on the acquisition of Golgi derived vesicles by *Chlamydia*. To test this, we used fluorescently labeled NBD-C6-ceramide as a marker for sphingomyelin uptake into the inclusion, as described previously [51]. HeLa cells were infected with *C*. *trachomatis* and grown for 22 hours. Cells were then treated with either 10 μM FLI-06, 5 mM 4PBA, or 3 μg/mL BFA for 1 hour, then incubated for 30 minutes with NBD-ceramide, followed by 1.5 hours back-exchange in the presence of ERES inhibitors or BFA. Fluorescence microscopy was used to assess mean fluorescence of the inclusions with different treatments. Similar to previous studies, treatment with BFA reduced ceramide uptake by 58.0% (Fig 8C). FLI-06 reduced uptake by about the same amount, 52.9%, indicating that although FLI-06 disrupts the Golgi, it does not affect chlamydial ceramide acquisition any more than BFA. Since BFA has no effect on IFU, it is unlikely that the reduction in IFU seen following FLI-06 treatment is due to the disruption of the Golgi. This is further supported by 4PBA having no discernible effect on ceramide uptake (Fig 8C).

### Depletion of ERES regulatory proteins disrupts *Chlamydia* growth

We next sought to determine the specific ERES and COPII associated proteins necessary for providing factors critical for chlamydial growth. Using RNAi, we knocked down the expression of Sec16A and TANGO1, proteins which are required for efficient COPII transport [41]. We additionally knocked down cTAGE5 and Sec12; Sec12 is the guanine exchange factor for Sar1 GTPase, which in turn regulates COPII vesicle formation [52]. Sec12 also interacts with cTAGE5 at ERES, and it has been shown that cTAGE5 can recruit Sec12 to COPII budding sites [53]. Bet3, a key component of the TRAPP complex that functions in COPII vesicle tethering and fusion at the ERGIC, was also knocked down [54].

**Fig 9:**
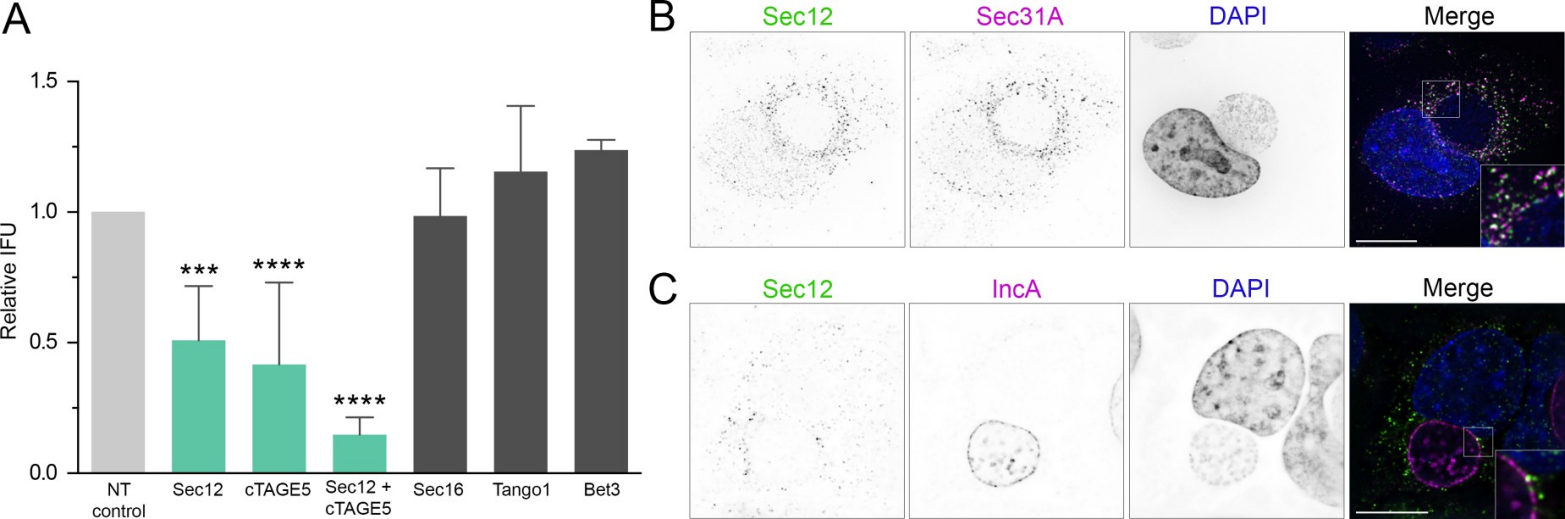
RNAi depletion of ERES regulatory proteins Sec12 and cTAGE5 disrupt *Chlamydia* growth. (A) Cells were treated with siRNA oligonucleotides and incubated for 48 hours, then infected with *C. trachomatis* L2. At 48 hpi, cells were lysed and infectious *Chlamydia* EB from each sample group were tested for IFU by infecting new cells. IFU values were compared to scramble siRNA treated, infected cells. Significance determined by one-way ANOVA with Dunnett’s multiple comparisons test, compared to control IFU. Bars, mean (SD);***, p < 0.001; ****, p < 0.0001; n ≥ 3. (B) Sec12 (anti-Sec12, green in merge) colocalizes with Sec31 (anti-Sec31A, purple in merge) in infected cells. Representative deconvolved merged z-series image. Scale bar = 16 μm. (C) Sec12 (anti-Sec12, green in merge) overlaps with IncA (anti-IncA, purple in merge) in a similar manner to Sec16 or Sec31. Single plane from deconvolved z-series image. Scale bar = 16 μm.

HeLa cells were transfected with siRNA oligos and incubated for 48 hr prior to infecting with *C*. *trachomatis*. At 48 hpi, cells were lysed and chlamydial IFU was quantified on fresh HeLa monolayers. Knockdown of Sec12 and cTAGE5 resulted in a 49.1% and 58.4% reduction in IFU, respectively (Fig 9A, S12 Fig). Simultaneous knockdown of both Sec12 and cTAGE5 reduced IFU by 85.2%. Surprisingly, Sec16A and TANGO1 depletion had no effect on IFU. Bet3 disruption also had no effect on IFU; however, this result was expected since Bet3/TRAPP functions downstream of ERES and COPII vesicle formation, as a tethering complex on the ERGIC and cis-Golgi. ERES proteins typically exhibit similar subcellular localizations, and we used immunofluorescence microscopy to determine if Sec12 had a similar distribution as other key COPII components during *Chlamydia* infection. Sec12 colocalized with Sec31 in infected cells (Fig 9B), and both proteins remained closely associated after treatment with FLI-06 or 4PBA (S13 Fig). Sec12 also overlapped with IncA in distinct punctae, similar to the overlap seen with Sec31 or Sec16 and IncA (Fig 9C). While it is unclear why Sec12 and cTAGE5 depletion affect IFU while Sec16 and Tango1 have no effect, it seems that *Chlamydia* may require a specific cargo or function of ERES rather than general COPII vesicular trafficking. It is also possible that there is redundancy in the roles of Sec16 and Tango1 during infection. Future efforts will need to resolve whether the basis of this interaction is to provide COPII vesicular cargo to inclusions, or to allow *Chlamydia* to interfere with an ERES-mediated process critical for infection.

## Discussion

Establishment and maintenance of the inclusion is critical to *Chlamydia*’s ability to infect and grow within host cells. Bioinformatic analysis of the *C*. *trachomatis* genome predicts over 50 type III secreted Inc transmembrane proteins [7,9,11]; however, little is known about the broad spectrum of host proteins that are recruited to the inclusion membrane. Recent proteomic studies have provided a major, initial snapshot of proteins that comprise mature inclusions [16] and that associate with exogenously expressed Inc proteins [15]. However, we still lack an understanding of host factors recruited to inclusions in their endogenous host cell setting, how the inclusion membrane interactome changes throughout the developmental cycle, and in particular what signaling pathways are critical for inclusion biogenesis. To advance knowledge in these important unexplored areas, we developed the APEX2 proximity-dependent biotinylation platform for *Chlamydia*, to allow type III mediated expression of Inc proteins, fused to APEX2, on the inclusion membrane. Using this approach, we obtained proteomic data for the dynamic recruitment of 452 host proteins to *C*. *trachomatis* inclusion membranes; this work strengthens existing proteomic datasets and additionally provides new insight into the *Chlamydia*–host interactions that shape the chlamydial intracellular inclusion niche [15,16]. APEX2 proximity dependent proteomics represents a powerful tool for investigating host–pathogen interactions. Previous work has demonstrated the efficacy of tagging IncA and IncF with APEX2 [17], and we urge the field to exploit this system to accelerate our understanding of the molecular functions of chlamydial type III secreted proteins.

A major finding of this study was the population of proteins assembled on early inclusions, as this subset is predicted to contain proteins important for regulating processes that shape inclusion biogenesis. Our data show that early inclusions, containing only a few bacteria, were enriched in proteins associated with the early secretory pathway and distinct cellular processes. In accordance with the need of *Chlamydia* to scavenge nutrients and energy from the host cell, proteins important for glycolysis were identified, including aldolases, transaldolase, transketolase, peroxiredoxins, and pyruvate kinase. Although these proteins were largely not identified at later time points, it is unclear if they are no longer in close proximity to the inclusion, or they represent a much smaller percentage of the inclusion membrane proteome at later times and were simply not detected. In addition, a large number of ER proteins were proximity labeled by IncB-APEX2, for example protein disulfide isomerase, calreticulin, calnexin, endoplasmin, apolipoprotein B-100, STIP1, and EMC10. Finally, members of the 14-3-3 protein family, serpins, and several cytoskeletal proteins—alpha-actinin-4, emerin, myosins, MYPT1, tropomyosins—were found to be in close proximity to inclusion membranes. We elected to perform a high throughput RNAi screen in order to test candidate proteins from the 8 hpi proteomic data. Depletion of many of these early association factors led to alterations in chlamydial growth, as measured by the production of IFU. This approach contained single siRNA oligonucleotides for each target, so follow-up studies would have to use independent sequences to ensure off-target effects did not affect IFU. Similarly, we did not assess the effect of knockdown on chlamydial entry into the cell so that could be another factor that impacted IFU. Among proteins recruited to early inclusions, RNAi knockdown of tropomyosins yielded unexpectedly large and disparate effects on bacteria growth. Knockdown of TPM2 enhanced IFU by over 2-fold, while knockdown of TPM4 resulted in close to 2 fold reduced IFU. Although tropomyosins affect actin filament stability, the different forms are thought to be functionally distinct.

Our study provides a third proteomic mapping of the inclusion membrane interactome, with each effort exploiting unique approaches and technological systems. We now have the opportunity to synthesize these proteomic datasets to derive a list of ‘high confidence’ interactions that were identified by all three proteomics studies and develop an understanding of multiprotein interactions that may occur with specific *C*. *trachomatis* Incs. Six proteins represented high confidence proteins recruited to early inclusions, three of which were previously shown to be recruited to inclusions: PDIA1 (PDI) [55], ASPH, MYPT1 [56], LRRF1, 14-3-3β [57], and LRC59. By 16 hpi, seven additional high confidence proteins were found to interact with inclusion membranes across all three proteomic studies: VAPA [49], VAPB [58], TMOD3, SNX1 [15,16], STX7 [16], BAP31, and CLINT1. Finally, 24 hpi *C*. *trachomatis* inclusions were consistently enriched with three additional host proteins: EGFR [59], 14-3-3ζ [60], and RTN4 [37]. The high frequencies with which these proteins have been independently demonstrated to associate with chlamydial inclusions strongly suggests that their enrichments in proteomic data sets are not merely a byproduct of high protein abundance in cells. Interestingly, BAP31, ASPH, and LRC59 are normally associated with the ER; LRC59 additionally interacts with FGF, and *C*. *trachomatis* EB have been shown to interact with FGFR on the cell surface [61]. Another lens with which to interpret inclusion interactome data is towards resolving protein networks that are intimately associated with a particular Inc protein. For example, our data revealed the recruitment of DYHC1, PCM1, MARE1, MAP1B, and PLK1, all annotated binding partners of DCTN2, to the inclusion membrane, and these interactions may be mediated through CT192, an Inc protein shown by AP-MS to also directly interact with DCTN2 [15]. Finally, our study indicated that chlamydial proteins constitute a small portion of the overall inclusion membrane proteome, as compared to host proteins. Only 11 Inc proteins were identified across all time points, suggesting that additional Incs may be expressed at lower levels; Incs may also be difficult to detect compared to many more abundant host proteins. Endogenous IncB colocalizes with CT101, CT222, and CT850 in microdomains on the inclusion membrane, but we did not detect these Incs in this study [26]. Co-immunoprecipitation with the microdomain-localizing Incs suggested that IncB does not bind CT101, CT222, or CT850, and colocalization by microscopy does not necessarily suggest that these Incs should be biotinylated by APEX more than other Incs that we detected. This may be due to mislocalization of IncB-APEX2 or these other microdomain-localized Incs may be low in abundance compared to the other Incs detected. The chlamydial protease CPAF has been shown to degrade certain host and chlamydial proteins post-cell lysis [62–64]. It is possible CPAF degraded some Incs and host proteins post-lysis and the degraded proteins were missed by mass spectrometry. IncB has no known binding partners, but many Incs have been shown to dimerize or bind other Incs [65]. This suggests another possibility that most Incs are sequestered into heteromeric complexes, such that even with induced overexpression, IncB based proximity labeling was unable to access the full repertoire of Incs present on the inclusion membrane. This outcome may also represent a limitation of the APEX2 approach for capturing all proximal proteins on inclusion membranes. It may be helpful in future experiments to do mass spectrometry analysis on a membrane fraction of cells following APEX2 labeling to enrich for membrane-integral proteins of the inclusion membrane. Some growth attenuation was observed for the IncB-APEX2 expressing strain, and IncB-APEX2 overexpression may perturb the distribution of endogenous Incs. Future efforts by the field should therefore focus on determining what extent of the inclusion membrane interactome is Inc-specific, and which Incs control the recruitments of host targets. The goal of this study was to exploit IncB-APEX2 to identify and compare proximal proteins to *C*. *trachomatis* inclusions across a temporal dimension, through generating quantitative mass spectrometry data at three discrete stages of *Chlamydia* and inclusion growth. An important limitation of this approach is that IncB-APEX labeled proteins were not cross-compared spatially against APEX2-tagged probes placed at other regions of host cells.

In this study, we also report the novel interaction between *C*. *trachomatis* inclusions and ER exit sites. Importantly, functional disruption of ERES cargo loading or specificity using chemical and genetic approaches resulted in major defects on inclusion and chlamydial growth. The ERES associated proteins Sec16, TANGO1, peflin, and TFG, were identified by IncB-APEX2 as inclusion membrane associated proteins and follow up investigations confirmed the recruitment of Sec16 and the COPII coat protein Sec31 to the cytosolic surface of inclusion membranes. Inclusion–ERES interactions seem to be distinct from previously described IncD- and IncV-mediated ER–inclusion membrane contact sites (MCS) [34,36,37,49,58], since the ER membrane protein CERT, which interacts with IncD at MCS, did not colocalize with ERES proteins. Localization of CERT and ER marker PDI was unaffected by inhibition of ERES using FLI-06 or 4PBA. In this regard, our findings strengthen the theme of inclusion membrane and ER interactions as playing important roles for inclusion biology and function during infection. Atlastin-3 and reticulon-4, which play roles in regulating ER morphology and structure [66,67], were also identified by our data and inclusion-MS [16]. In addition to ERES and COPII components, inclusion membrane interactions contain ERGIC-53 and VIP36 [16], two homologous proteins which are primarily localized to the ERGIC and function to regulate COPII vesicle fusion, and ER-ERGIC-Golgi syntaxins 5 and 18 [16]. We propose *C*. *trachomatis* inclusions intimately interact with COPII mediated ERES to ERGIC vesicular traffic.

Reasons for the importance of Sec12 and cTAGE5, but not Sec16 or Tango1, in the interaction between ERES and inclusion membranes are at this time unclear. Recent work has shown that nutrient starvation induced Sec12 and cTAGE5 relocation to the ERGIC followed by generation of autophagosome membrane precursors [68,69]. There are likely many other roles of ERES that have not yet been described, and the molecular mechanisms of ERES and ERGIC regulation are incompletely understood. FLI-06 blocks cargo recruitment to ERES, but unfortunately there are no known proteins involved in this step of COPII trafficking. The targets of 4-PBA, the p24 (also called TMED) family, would be very interesting to study in the context of *Chlamydia* infection, especially since five of these proteins were found by AP-MS or inclusion-MS [15,16]. These experiments may prove challenging, however, as there are 11 members and they are thought to have functional redundancy.

Collectively, the functional and proteomic data show that complete developmental growth of *Chlamydia* requires efficient COPII vesicle production from ERES throughout the developmental cycle. One attractive nutritive benefit for *Chlamydia* is the acquisition of lipids, as supported by evidence that chlamydiae acquire phosphatidylcholine and other phospholipids from host cells [70,71], recruit lipid droplets to inclusions [72], redirect cholesterol to inclusions [73], and require functional host fatty acid synthesis machinery [72,74]. Much of our understanding for ERES secretory mechanisms has come from studying the assembly of large cargo into COPII vesicles; for example procollagen, pre-chylomicrons, and very low-density lipoproteins [75]. For export of these bulky cargo, cTAGE5 and TANGO1 are essential. In our system, cTAGE5 disruption impaired IFU production, whereas TANGO1 knockdown had no discernible effect. An explanation for these findings is elusive, notably because only a preliminary functional characterization of these regulatory proteins exists. Interestingly, cTAGE5 and the TANGO-related gene MIA2 can form a fusion protein (TALI) which binds TANGO1 and facilitates the recruitment of apolipoprotein B containing lipid complexes to ERES [76]. Based on our proteomic data, apolipoprotein B is recruited to inclusion membranes throughout the developmental cycle.

## Methods

### Antibodies and reagents

All reagents were purchased from Thermo Fisher Scientific (Rockford, IL) unless otherwise noted. Primary antibodies used in this study and their catalog numbers: Sec12 (Western blot, PA5-53125), Sec12 (Immunofluorescence, gift from Kota Saito), cTAGE5 (PA5-29515), Sec31A (Cell Signaling, Danvers, MA; 13466S), Bet3 (TRAPPC3; PA5-55841), Sec16A (Bethyl Laboratories, Montgomery, TX; A300-648A-M), TANGO1 (MIA3; Millipore Sigma, St. Louis, MO; SAB2700012), GM130 (R&D Systems, AF8199SP), IncA (gift from Daniel Rockey), CT223 (gift from Daniel Rockey), MOMP (Virostat, Westbrook, ME; 1621), Flag (Millipore Sigma; F1804). Secondary antibodies used: Donkey anti-Rabbit HRP, Donkey anti-Goat Alexa 594, Goat anti-Mouse DyLight 594, Goat anti-Rabbit Alexa 488, Streptavidin Alexa 488.

### Cell culture and *Chlamydia* infections

HeLa 229 cells (ATCC) or McCoy cells (obtained from Walt Stamm) were grown in Roswell Park Memorial Institute 1640 (RPMI; Gibco) medium supplemented with 10% fetal bovine serum (HyClone) and 2 mM L-glutamine (HyClone) at 37°C, 5% CO_2_. *Chlamydia trachomatis* LGV L2 434/Bu was grown in HeLa 229 cells for 48 hours, then infected cells scraped into sucrose phosphate buffer (5 mM glutamine, 0.2 M sucrose. 0.2 M phosphate), lysed using bead bashing, and cell debris cleared by centrifugation at 300 × g for 10 minutes. Supernatant containing *Chlamydia* aliquoted and stored at -80°C. HeLa cells were infected with *Chlamydia* diluted in Hank’s buffered salt solution (HBSS; Gibco) at an MOI ~1 for 2 hours at room temperature. Cells were washed with HBSS, then incubated at 37°C in RPMI.

### Plasmid constructs and generation of transformed *Chlamydia* strains

Plasmids were isolated using the Qiaprep Spin Miniprep kit or the HiSpeed Plasmid Midi kit (Qiagen, Germantown, MD).

*Chlamydia* plasmid expressing IncB-APEX2 fusion was made in a modified version of the pASK-GFP parent vector provided by Scott Hefty [29], where GFP was replaced with IncB-APEX2 [19] and the mKate2 sequence was removed. The plasmid containing the APEX2 sequence was a gift from Scott Hefty. Plasmid grown in dam- *E*. *coli* (C2925; NEB, Ipswich, MA) prior to *Chlamydia* transformation. Plasmid transformed strains of *C*. *trachomatis* L2 were generated using established procedures [24]. Three different dilutions (undiluted, 1:2, 1:10) of *C*. *trachomatis* L2 stocks were made in 50 μL calcium chloride buffer (20 mM Tris pH 7.4, 100 mM CaCl2). In the same buffer, 3 ug plasmid was diluted to 50 μL and added to diluted *Chlamydia*. The 100 μL *Chlamydia*–plasmid mixture was incubated for 30 minutes at room temperature. McCoy cells were trypsinized and diluted to a final concentration of 4 x 10^7^ cells/ml in calcium chloride buffer. After 30-minute incubation, 100 μL diluted McCoy cells were added to *Chlamydia* and DNA mixture, and incubated for 20 minutes. In a 6 well plate, 100 μL McCoy cells, plasmid, and *Chlamydia* mixture were added to 2 mL medium. At 12–15 hours post infection, cells were washed and medium added containing 2.5 U/mL Penicillin G (Millipore Sigma) and 1 ug/mL cycloheximide. Cells were incubated for 48 hours, then *Chlamydia* passaged onto fresh McCoy cells. After first passage, cells were grown in medium containing 10 U/mL Penicillin G and 1 ug/mL cycloheximide. Transformants were passaged at least 2 more times, every 48 hours, until inclusions were apparent. Stocks of transformed *Chlamydia* were frozen at -80°C as described above. Transformants were not plaque purified to obtain a clonal population but expression of Inc-APEX2 fusions was assessed by microscopy to check for uniform expression and localization following induction with ATc.

Mammalian plasmid pmScarlet-CERT was made in the pEGFP-N1 backbone, with Gibson Assembly used to replace EGFP with mScarlet. mScarlet was amplified from the pmScarlet-C1 plasmid, which was a gift from Dorus Gadella (Addgene plasmid # 85042) [77]. CERT was inserted at the N terminus of mScarlet using the XhoI and AgeI restriction sites. The pmClover3-PDI plasmid was made in the pEGFP-N1 backbone. Gibson Assembly was used to replace EGFP with mClover3, and PDI was inserted at the N terminus of mClover3 using Gibson Assembly. mClover3 was amplified from pKanCMV-mClover3-18aa-actin, which was a gift from Michael Lin (Addgene plasmid # 74259) [78]. Plasmid pmGFP-Sec16L was a gift from Benjamin Glick (Addgene plasmid # 15776) [79].

### Biotin-phenol labeling in live cells

Biotin-phenol labeling of *Chlamydia* infected cells was adapted from described a protocol [80]. HeLa cells were grown in 8 well chamber slides (for immunofluorescence detection of biotinylation; Nunc Lab-Tek), or a T75 flask (for western blot and mass spectrometry), then infected with *C*. *trachomatis* L2 expressing IncB-APEX2 at an MOI of approximately 1. Cells were incubated in RPMI containing 1 ng/mL anhydrotetracycline (ATc; Acros Organics, New Jersey) and 1 ug/mL cycloheximide (Gold Biotechnology, St. Louis, MO). At 30 minutes prior to time point, 2.5 mM biotin-phenol (Iris Biotech, Marktredwitz, Germany) in RPMI was added to cells. At each time point, 30% hydrogen peroxide diluted to 100 mM working stock in Dulbecco’s phosphate-buffered saline (DPBS; Gibco) was added to cells at a final concentration of 1 mM and allowed to incubate with gentle rocking for 1 minute at room temperature. Labelling solution was aspirated, and cells were rinsed 3 times in quenching solution (10 mM sodium ascorbate, 5 mM Trolox, 10 mM sodium azide in DPBS). For immunofluorescence, normal fixation and staining protocols were followed. For western blot and mass spectrometry, cells were scraped into quenching solution and centrifuged for 5 minutes at 3,000 x g, 4°C. Cells were lysed by resuspending in ice cold RIPA buffer (50 mM Tris, 150 mM NaCl, 0.1% SDS, 0.5% sodium deoxycholate, 1% triton X-100, pH 7.5) with Halt protease inhibitor cocktail (Pierce) and quenchers (10 mM sodium ascorbate, 5 mM Trolox, 10 mM sodium azide). Lysate was incubated for 2 minutes on ice, then sonicated 3 x 1 second at 20% amplitude, and clarified by centrifugation for 10 minutes at 15,000 x g, 4°C. Part of the lysate was reserved for western blot, and remaining lysate for each sample was snap frozen until mass spectrometry processing.

### Protein purification for mass spectrometry

Protein concentrations for individual samples were measured by BCA. The samples were normalized to 2 mg/mL for enrichment. Labeled protein lysates were enriched with streptavidin agarose resin (Thermo Fisher Scientific, Rockford, IL). The resin was prepped for enrichment by placing the resin in a Bio-Rad chromatography column (Bio-Rad, Hercules, CA) on a vacuum manifold. The resin was washed with 0.5% SDS in PBS (1 mL, repeat 2×), 6 M urea in 25 mM ammonium bicarbonate (NH_4_HCO_3_) (1 mL, repeat 2×), and PBS (1 mL, repeat 4×). The resin was transferred to 4 mL cryovials using two 1 mL aliquots of PBS. An additional 0.5 mL of PBS was added to each tube followed by 1000 μg of protein (in 1.2% SDS in PBS). The total volume of each tube was set to 3.0 mL, giving a final SDS concentration of 0.2%. Tubes were rotated end over end for 4 hr at room temperature. Following streptavidin capture of biotinylated proteins, the solution was transferred into the Bio-Rad columns, and the solution was removed. The resin was washed with 0.5% SDS in PBS (1 mL, repeat 2×), 6 M urea in 25 mM NH_4_HCO_3_ (1 mL, repeat 2×), Milli-Q water (1 mL, repeat 2×), PBS (1 mL, repeat 8×), and 25 mM NH_4_HCO_3_ (1 mL, repeat 4×). The enriched resin was transferred to sealed 1.5 mL tubes using two 0.5 mL aliquots of 25 mM NH_4_HCO_3_. Samples were centrifuged at 10,500 x g, and the supernatant was discarded. 6M Urea was added to the resin for each sample followed by 100 mM TCEP (20uL) and place on a thermomixer for 30 min (1200 rpm at 37°C). After the samples were reduced, 200 mM iodoacetamide (20uL) was added to alkylate the proteins. The resin was placed back on the thermomixer for 45 min (1200 rpm at 50°C) and covered in foil. Following alkylation, the samples were returned to the Bio-Rad column and rinsed with PBS (1mL, repeat 8×) and 25 mM NH_4_HCO_3_ (1 mL, repeat 4×). The enriched resin was transferred to sealed 1.5 mL tubes using two 0.5 mL aliquots of 25 NH_4_HCO_3_. Samples were centrifuged at 10,500 x g, and the supernatant was discarded. Enriched biotinylated proteins were prepared for LC−MS/MS analysis. 25mM NH_4_HCO_3_ (200 μL) was added to the resin for each sample, along with trypsin solution. Resin solutions were placed on the thermomixer at 37°C set at 1200 rpm set for overnight. Following trypsin digestion, the tryptic peptides were collected, and the resin washed once with 25 mM NH_4_HCO_3_ (150 μL). Volatiles were then removed from the combined tryptic peptide supernatant using a speed vacuum. The dried peptides were reconstituted in 25 mM NH_4_HCO_3_ (40 μL) and heated for 10 min at 37°C with mild agitation. To remove any solid particulates, samples were centrifuged at 53,000 x g for 20 min at 4°C. From each ultracentrifuge vial was removed 25 μL for MS analysis. Samples were stored at −20°C until analysis.

### Mass spectrometry and bioinformatic analysis of proteomic data

Tryptic peptides from enriched proteins were separated using in-house reverse-phase resin columns by LC and analyzed on a Thermo Fisher Velos Orbitrap MS as described previously [81]. Instrument data was acquired for 100 min, beginning 65 min after sample injection into the LC. Spectra was then collected from 400–2,000 *m/z* at a 100k resolution, following by data-dependent ion trap generation of MS/MS using the top six most abundant ions, a collision energy of 35%, and a dynamic exclusion time of 30 s for discriminating against previously analyzed ions. MS/MS spectra were searched using the MSGF+ algorithm and a tag-free quantitative accurate mass and time (AMT) tag approach for subsequent unique peptide to protein mapping using LC-MS peak feature detection, as described previously [82] with the following modifications. MS/MS spectra were searched against the following FASTA files for *Chlamydia* and *Homo sapiens*: *Chlamydia trachomatis* serovar L2 434 Bu pL2Plasmid 2018-01-05 and *Homo sapiens* Uniprot SPROT 2017-04-12. Peptide MS/MS features from each MS dataset were filtered on an FDR of less than or equal to 1%. Unique peptides, requiring a minimum of six amino acids in length, were filtered using an MS-GF threshold of ≤ 1 ×10^−9^, corresponding to an estimated false-discovery rate (FDR) <1% at a peptide level. Resulting relative peptide abundances, in replicate across 48 biological samples including control samples, were log transformed and the data was processed for quality control. Elimination of statistical outliers were confirmed using a standard Pearson correlation at a sample level [83]. Parameters for removing inadequate data for qualitative statistics required a minimum of two observations for a peptide across all groups to be compared quantitatively or identified in at least half the biological replicates for a given condition group using previously described methods [84]. Peptides were normalized using median centering, adjusting for overall differences in abundances across samples. Statistical comparisons were made between control and biotinylated groups at each time point and evaluated for quantitative differences using a standard 2-sample t-test and a qualitative difference (presence/absence markers) g-test. Statistical test used for each protein is shown in S2 Table. Additional evaluations were done in a similar manner for comparison of conditions across time. The mass spectrometry proteomics data have been deposited to the ProteomeXchange Consortium via the PRIDE partner repository [85] with the dataset identifier PXD012494 and 10.6019/PXD012494.

For KEGG pathway analysis, UniProt identifiers of proteins were uploaded into InnateDB pathway analysis, then processed using the pathway overrepresentation analysis tool. The recommended settings were used for the analysis: hypergeometric algorithm, Benjamini Hochberg *p* value correction method. Only pathways with *p* < 0.05 after Benjamini Hochberg correction were listed.

To assign subcellular localization ontologies to proteins, the Human Protein Atlas database (version 18) was used [33]. Subcellular localization data for all proteins was downloaded, and data for proteins in IncB-APEX2 data set was extracted. Only subcellular locations with enhanced, supported, or approved reliability scores were used. Proteins with more than one annotated location were counted for each location. Highly similar categories were condensed, for example nuclear speckles, nucleoli, nucleoli fibrillar center, and nucleoplasm were all counted as nuclear lumen.

Network analysis of APEX2 proteins was done using annotations from StringDB (version 10). Only interactions with evidence from experiments or databases were considered, with a confidence score of at least 0.700 (high confidence). Proteins with no interaction partners were excluded. Annotated interactions were downloaded for the proteins each time point, and this data was compiled into a list of all interactions at different time points using Microsoft Excel. To make the graph more readable, proteins involved in translation were excluded. This data was imported into RStudio software and plotted using the tidygraph package [86]. Code details are provided in the supplemental methods. Graph layout was arranged using the Fruchterman Reingold algorithm, and subsets of closely interacting protein communities were detected using the Louvain method. General categories of some of the protein communities were assigned based on similarities between the UniProt descriptions of proteins in that particular community. If there were only 2 proteins in a group, or there was no consensus between the UniProt descriptions, no category was assigned.

To compare to the AP-MS [15] and inclusion-MS [16] protein lists, full data sets were obtained from the supplemental data of each study. Proteins were matched based on UniProt identifiers, and all three data sets were combined using RStudio and Microsoft Excel (S1 Table). For the AP-MS data, MIST scores are listed (closer to 1 is better). All Inc proteins that were found to interact with the host protein were listed, with the MIST score from the first listed Inc retained. For APEX2 and inclusion-MS, *p* values are listed.

### siRNA and plasmid transfections

For siRNA transfections, HeLa cells were plated in 24 well or 96 well plates to 60–80% confluence. For 24 well plates, 50 μL Opti-MEM (Gibco) medium containing 5 pmol siRNA oligonucleotides and 1.5 μL Lipofectamine RNAiMAX was added to each well. Following transfection, cells were incubated for 48 hours at 37°C prior to infection or protein analysis by western blot. For 96 well plate experiments, knockdowns were done in duplicate, with 10 μL Opti-MEM containing 1 pmol siRNA oligonucleotides and 0.3 μL Lipofectamine RNAiMAX added per well. Oligonucleotides were purchased from Dharmacon, sequences and catalog numbers listed in supplemental methods. Dharmacon siGenome smartpools of 4 different oligonucleotide sequences were used for Sec16, Tango1, and Bet3 knockdowns. Dharmacon siGenome individual oligonucleotides were used for all other knockdowns.

For mammalian plasmid transfections, HeLa cells were plated in 4 well chamber slides (Nunc Lab-Tek), and infected with *C*. *trachomatis* L2. Immediately after infection, each well was transfected with 50 μL Opti-MEM containing 500 ng plasmid and 1.5 μL Lipofectamine 2000.

### Quantitative PCR analysis

For qRT-PCR confirming siRNA knockdown, HeLa cells were plated in 24 well plates, and transfected with siRNA oligos as described above. Cells were incubated for 48 hours, then cell pellets were frozen until RNA extraction.

For qRT-PCR measuring ER stress, HeLa cells were grown in 6 well plates and infected with *C*. *trachomatis* for 20 hours. At 20 hpi, 10 μM FLI-06, 5 mM 4PBA, 1 μM thapsigargin or DMSO was added in fresh medium. At 24 hpi, cells were trypsinized and cell pellets frozen until RNA extraction.

RNA was extracted using the Qiagen RNeasy mini kit (Qiagen). cDNA was made using the iScript cDNA synthesis kit (Bio-Rad). qRT-PCR reactions were set up with SsoAdvanced Universal SYBR Green Supermix (Bio-Rad) and run on the StepOnePlus Real-Time PCR system (Applied Biosystems, Foster City, CA). GAPDH was used as the housekeeping gene, and the ΔΔCT method was used to calculate relative expression. Primer sequences are listed in the supplemental methods.

For *Chlamydia* genome copy number analysis HeLa cells were infected with *C*. *trachomatis* for 24 hours, then 10 μM FLI-06, 5 mM 4PBA, 0.5 μg/mL chloramphenicol, or DMSO was added in fresh medium. At 48 hpi, cell pellets were trypsinized, centrifuged at 300 x g for 5 minutes, then *Chlamydia* genomic DNA was extracted using the DNeasy Blood and Tissue kit (Qiagen). qPCR was done with primers to GroEL2 (sequences listed in supplemental methods).

### Immunofluorescence and live cell microscopy

For immunofluorescence microscopy, cells were rinsed in HBSS, then fixed for 5 minutes in ice cold methanol (Images with anti-Sec12 only, for methanol fixation permeabilization was skipped) or 15 minutes in 3.7% paraformaldehyde in HBSS. Cells were rinsed 2 times in HBSS, then permeabilized with 0.5% triton X-100 for 15 minutes, and blocked in 1% bovine serum albumin (BSA) in PBS for 20 minutes. Samples were incubated with primary antibodies for 1 hour at room temperature in blocking buffer, then rinsed 3 times in blocking buffer, then incubated 45 minutes at room temperature with secondary antibodies in blocking buffer. DAPI was used to stain DNA, and added during the secondary antibody step. For biotin labeling, Streptavidin-Alexa 488 was incubated for 30 minutes at room temperature. For imaging of live cells, media was replaced with HBSS before imaging. Cells were imaged on a Nikon Ti-E inverted microscope and images were captured on a Hamamatsu camera controller C10600. Images were processed using Volocity software (PerkinElmer, Waltham, MA).

Quantification of Sec16A and Sec31A distribution around the inclusion in infected cells was analyzed using CellProfiler (Version 3.1.8). Full pipeline available in S1 File. Representative images from certain steps in pipeline shown in supplemental methods. HeLa cells were plated on glass and infected with *C*. *trachomatis* L2. Cells were fixed and stained as described above, with anti-Sec16A or anti-Sec31, anti-IncA, and DAPI. 20x images were taken using the Nikon Ti-E inverted microscope, as described above. Separate channels of DNA, Sec31A or Sec16A (will refer to as ERES channel), and IncA were loaded into the pipeline. Color images were converted to grayscale, ERES channel intensity was rescaled to make different image batches more comparable. IdentifyPrimaryObjects was used to identify inclusions, nuclei, and ERES. Inclusions were sometimes identified as nuclei in the DAPI channel, so these inclusions were subtracted from the nuclei objects using IdentifyTertiaryObjects. ERES objects in infected cells were retained using RelateObjects, while other ERES objects were not analyzed further. ERES in infected cells were merged using SplitOrMergeObjects so that all ERES associated with a certain inclusion are counted as one object. Inclusions and their associated ERES were counted using DisplayDataOnImage so any measurements could be associated with a certain inclusion. Inclusion perimeter was isolated by shrinking the inclusion objects by 2 pixels using ExpandOrShrinkObjects, then subtracting the shrunken inclusion from the full-size inclusion using MaskObjects. This left objects that were a 2-pixel wide perimeter around the inclusion. Next, any region of the inclusion perimeter that overlapped the ERES objects was masked. MeasureObjectSizeShape was used to measure the size of the inclusion perimeter, as well as the inclusion perimeter left after subtracting any portions that were overlapping ERES. This data was exported to Microsoft Excel, where the length of the inclusion perimeter that didn’t overlap ERES was subtracted from the full perimeter length. This value represents the length of the inclusion that overlaps ERES. This number was divided by the full length of the inclusion to get the proportion of the inclusion that is closely associated with concentrated ERES.

Sec31A punctae overlapping the inclusion were counted manually using Volocity software. Spots that overlapped with IncA, or were touching IncA were counted as on the inclusion membrane. Region where inclusion is adjacent to nucleus was excluded from analysis. Images used for counting were 60x magnification, deconvolved z-series. Planes were examined individually to ensure overlap was in the same z dimension. At least 20 inclusions in control and FLI-06 treated cells were analyzed, for each of two independent experiments.

Ceramide trafficking to the inclusion was measured as previously described, with slight modifications [87]. HeLa cells were infected with *C*. *trachomatis* at an MOI < 1 for 22 hours. Warm media containing 10 μM FLI-06, 5 mM 4PBA, 3 μg/mL BFA, or DMSO was added to the cells for 1 hour at 37°C. NBD-ceramide was complexed to BSA by adding 10 μM NBD-ceramide to HBSS with 0.7% fatty acid free BSA (Millipore Sigma) and vortexing thoroughly. After 1-hour incubation with inhibitors, cells were rinsed 1 time with HBSS, then NBD-ceramide/BSA was added and cells incubated for 30 minutes at 4°C. Cells were rinsed two times in HBSS, then incubated for 1.5 hours at 37°C in serum free media with 0.7% fatty acid free BSA with same inhibitors as before. After 1.5 hours, stained nuclei using Hoechst and took images with fluorescence microscope. Analyzed 5 microscopy fields per condition using Volocity software. For each field, made 30 identical circles that fit inside nuclei to get mean background fluorescence, outlined 20 inclusions (100 total per replicate) by hand to measure mean fluorescence inside inclusion. Outlines were done in Hoechst channel only to avoid bias, measurements were taken from NBD channel only. Background fluorescence was subtracted from inclusion fluorescence to get measurements shown in graph. Data compiled from three independent experiments.

For quantification of CERT and PDI association with the inclusion following treatment with ERES inhibitors, HeLa cells were plated in glass chamber slides, transfected with pmScarlet-CERT or pmClover-PDI using Lipofectamine 2000, following manufacturer’s instructions. Cells were then infected with *C*. *trachomatis* L2 at an MOI ~1. At 20 hpi, warm media containing 10 μM FLI-06, 5 mM 4PBA, 3 μg/mL BFA, or DMSO was added to the infected cells. At 24 hpi the cells were fixed and stained with anti-IncA and DAPI. 60x z-series images were acquired and deconvolved. Images were cropped to focus on specific inclusions, and all planes with IncA out of focus were removed and not included in image analysis. Images were all processed identically using an ImageJ macro (available in supplemental methods). Images were separated by channel and channels were automatically thresholded using the Moments method (CERT, IncA), or Triangle method (PDI) to get a binary image of each channel. These binary images were analyzed using the Coloc 2 plugin to obtain the manders M1 and M2 coefficients. Note that Coloc 2 has its own thresholding algorithm that did not work for our images, so we only looked at the M1 and M2 for imported images since they were thresholded before being run through Coloc 2. These coefficients were exported to Prism for statistical analysis. For PDI, since the majority of the protein is not associated with the inclusion only the manders M2 corresponding to the percentage of IncA that colocalized with PDI. For CERT, we only reported the manders M1 which represents the percentage of CERT (within the field) that colocalizes with IncA.

### Western blot analysis

For western blot analysis of biotinylated proteins [80], 6x protein loading buffer was added to lysates, samples were boiled for 5 minutes, followed by cooling on ice. 15 μl lysate was loaded and run on 10% SDS gel in Tris running buffer by SDS-PAGE. Proteins were transferred from gels onto Immobilon PVDF membrane (Millipore Sigma) with a Pierce G2 fast blotter in Pierce 1-Step transfer buffer, then blocked with 3% BSA in TBST overnight at 4°C. Blots were incubated with streptavidin-HRP in 3% BSA in TBST for 30 minutes at room temperature, washed 4 x 5 minutes in TBST, incubated with chemiluminescent substrate (Li-Cor 92695000, Lincoln, NE) for 5 minutes, and imaged using a C-DiGit blot scanner (Li-Cor). For western blot analysis of RNAi knockdowns, cell pellets were lysed in ice cold RIPA buffer (50 mM Tris, 150 mM NaCl, 0.1% SDS, 0.5% sodium deoxycholate, 1% triton X-100, pH 7.5) with Halt protease inhibitors (Pierce) for 30 minutes on ice, with vortexing every few minutes. Lysates were centrifuged for 20 minutes at 14,000 x g, and supernatant added to 4x laemmli buffer (Bio-Rad, Hercules, CA). Lysates were run by SDS-PAGE on 5–15% or 5–20% mini-PROTEAN TGX stain free gels (Bio-Rad), transferred to Immobilon PVDF membrane (Millipore Sigma), blocked for 1 hr in 5% milk-TBST, and labeled with antibody and digitally imaged as described above.

### Inclusion forming unit analysis

Infected HeLa cells were lysed at 48 hpi by incubating in water for 20 minutes followed by pipetting to disrupt cells. Serial dilutions of lysate were plated onto fresh HeLa monolayers in a 96 well plate. At 24 hpi, cells were fixed and stained with DAPI and an anti-MOMP antibody. Using immunofluorescence microscopy, 10–15 fields per well were taken at 20x magnification. Inclusions and nuclei in each field were counted using the Fiji distribution of ImageJ [88]. The macro used to count inclusions and nuclei in images is provided in supplemental methods. The overall percentage of infected cells was used to compare IFU between conditions, and the relative IFU calculated for each experiment by comparing to control. For FLI-06 experiments, HeLa cells in 24 well plate were infected with *C*. *trachomatis* L2 at an MOI around 1. FLI-06 was resuspended in DMSO to a concentration of 20 mM and frozen at -80°C until use. For treating cells, FLI-06 in DMSO was diluted 1:2000 in normal cell medium to make a final concentration of 10 μM FLI-06 and serial dilutions were used to make medium with 5 μM and 1 μM FLI-06. At 2.5, 18, 24, or 40 hours post infection, medium was aspirated from different wells in the plate and replaced with pre-warmed medium containing three different FLI-06 concentrations. Control well medium was aspirated and replace with fresh pre-warmed medium. For the 18-hour time point, two wells per concentration were prepared, one of which was aspirated and washed with HBSS at 24 hpi, then incubated in fresh medium. Since the stability of FLI-06 was unknown, medium in wells treated at 2.5 hpi was refreshed at 24 hpi, with identical concentrations of FLI-06. At 48 hpi, primary infection was imaged on microscope and 10–15 brightfield images were taken per well at 20x magnification and inclusion diameter was measured using Volocity software. After images were taken, cells were lysed for IFU assay as described above. IFU was adjusted based on number of inclusions per field in primary infection due to differences in cell growth with the inhibitor.

### Statistical analysis

Statistical analysis was performed using GraphPad Prism software. Comparisons between IFU, inclusion diameter, or genome copy number were analyzed using one-way ANOVA with Dunnett’s test for multiple comparisons. Comparison for number of Sec31 punctae on the inclusion membrane with or without FLI-06 treatment were analyzed using an unpaired t-test with Welch’s correction. Ceramide uptake after treatment with inhibitors was analyzed using a one-way ANOVA with Tukey’s multiple comparisons test. ER stress in infected and uninfected cells after treatment with inhibitors was analyzed using a two-way ANOVA with Dunnett’s multiple comparisons test. Quantification of CERT or PDI association with inclusion was analyzed by one-way ANOVA. CERT was additionally analyzed with Dunnett’s multiple comparisons test. Graphs indicate mean and standard deviation, statistical significance is indicated as follows: *, *p* < 0.05; **, *p* < 0.01; ***, *p* < 0.001; ****, *p* < 0.0001.

## Supporting information

S1 Fig Overexpression of different Inc-APEX2 fusions causes unique localizations and effects on inclusion growth. (A) HeLa cells were infected with *C*. *trachomatis* transformed with plasmids containing several known Incs fused to flag-APEX2. 1 ng/mL ATc was added at the start of infection, cells were fixed at 24 hpi and stained with anti-flag (white) and DAPI (blue). Images shown are single planes of deconvolved z-series. Scale bars = 16 μm. (B) Cells were infected with *C*. *trachomatis* transformed to express IncC-APEX2 with a flag tag, cells were treated as described in A. IncC-APEX2 was sometimes distributed evenly around the inclusion (top images), while in other inclusions it had a microdomain localization pattern (bottom images). Scale bars = 16 μm. (C) Cells were infected and fixed as described in A, and inclusion diameter was measured. Incs listed are referring to the Inc fused to APEX2 in the *Chlamydia* transformant tested. Each dot represents one inclusion, red line represents mean diameter with SD in black. Mean diameter values in left to right order were 9.40 μm, 10.07 μm, 8.82 μm, 8.16 μm, 6.71 μm.
(TIF)

S2 Fig IncB-APEX2 overexpression requires ATc and does not disrupt endogenous IncA or CT223 localization. HeLa cells were infected with *C*. *trachomatis* transformed with tet inducible, flag tagged, IncB-APEX2 plasmid. Cells were grown in normal conditions or with 1 ng/mL ATc (rows labeled +ATc). At 24 hpi, cells were fixed and stained with anti-flag, anti-IncA (A), or anti-CT223 (B) antibodies and DNA was labeled with DAPI. Images are 20x magnification (Top two rows of A and B, scale bars = 32 μm) or single plane from 60x deconvolved z-series (Bottom two rows of A and B, scale bars = 16 μm). (C) Same samples as described in A, B, plot of inclusion diameters with or without 1ng/mL ATc. Each dot represents one inclusion, measurements taken from two independent experiments. Significance determined by two-tailed Mann-Whitney test, red line shows mean (SD); ****, *p* < 0.0001. Mean diameter for untreated inclusions was 11.22 μm and 9.40 μm for inclusions treated with 1 ng/mL ATc.
(TIF)

S3 Fig mRNA expression of targets that alter Chlamydia IFU by at least 1.5 fold is reduced following siRNA treatment. HeLa cells were transfected with siRNA oligos corresponding to the genes listed in graph or non-targeting control. Relative expression was measured using qRT-PCR using the ΔΔCT method at 48 hours post transfection. Order corresponds to effect on IFU, from left to right (not including control) is from highest reduction in IFU to most increased IFU.
(TIF)

S4 Fig Histogram of fraction of inclusion membrane with concentrated Sec16A or Sec31A associated. HeLa cells were infected with *C*. *trachomatis* L2 and fixed at 24 hpi. Cells were stained with anti-IncA and either anti-Sec16A or anti-Sec31A and 20x images were taken. Concentrated regions of ERES marked by Sec16A (A) or Sec31A (B) were outlined using CellProfiler, complete description of image processing is in supplemental methods [44]. Inclusion perimeters were also outlined using CellProfiler, and the fraction of inclusion perimeter that overlapped the outlined concentrated ERES was calculated. Each inclusion was calculated individually, graph shows data from two independent trials with at least 200 inclusions measured per trial. Percentage refers to percentage of inclusions with specified fraction overlap.
(TIF)

S5 Fig Sec16A and Sec31 associate with the inclusion membrane early in infection. HeLa cells infected with *C*. *trachomatis* L2 were fixed at 14 hpi and stained with anti-Sec16A (A) or anti-Sec31A (B), anti-IncA, and DAPI. Top rows of A and B are deconvolved and merged z-series images; bottom rows are single deconvolved planes. Scale bars = 10 μm.
(TIF)

S6 Fig Sec16 is recruited to C. trachomatis inclusions in living cells, and FLI-06 abrogates the association. HeLa cells were transfected with a Sec16-GFP plasmid and infected with mCherry expressing *C*. *trachomatis* L2. DNA was labeled with Hoechst and cells were imaged live at 24 hpi. In top row, Sec16-GFP shows a similar localization to antibody staining (Fig 5A), with an enrichment near the inclusion membrane. Bottom row shows cells treated with 10 μM FLI-06 from 20–24 hpi, resulting in diffuse Sec16-GFP punctae throughout the cell. Scale bars = 16 μm, images are deconvolved merged z-series.
(TIF)

S7 Fig ERES are distributed around the inclusion even during Golgi disruption. HeLa cells were infected with *C*. *trachomatis* and treated with either DMSO or 3 μg/mL BFA from 20–24 hpi. Cells were fixed at 24 hpi and stained with GM130 (anti-GM130, purple in merge) to mark the Golgi, Sec31A (anti-Sec31A, green in merge) to mark ERES, and DAPI for DNA (blue in merge). Scale = 16 μm, images are deconvolved merged z-series.
(TIF)

S8 Fig ERES marker Sec16A is more diffuse following FLI-06 treatment. HeLa cells were infected with *C*. *trachomatis* and incubated with FLI-06 from 20–24 hpi, then fixed and processed for immunofluorescence. Sec16A (anti-Sec16A, green in merge), IncA (anti-IncA, purple in merge), and DNA (DAPI, blue in merge) were labeled, showing an altered localization of ERES in the presence of FLI-06. Scale = 16 μm, images are deconvolved merged z-series.
(TIF)

S9 Fig Inclusion growth impacted following FLI-06 treatment. Brightfield images at 20x magnification of infected cells treated with FLI-06 starting at 18 hpi, at indicated concentrations, and measured at 48 hpi. At 10 and 5 μM FLI-06, inclusion morphology appears smaller with bacteria less uniformly distributed, compared to the 1 μM or untreated cells. Arrows indicate inclusions.
(TIF)

S10 Fig Quantification of CERT and PDI association with the inclusion after treatment with ERES inhibitor or BFA. HeLa cells were transfected with plasmids pmScarlet-CERT (A) or pmClover-PDI (B) then infected with C. trachomatis L2 and fixed at 24 hpi. Cells were stained with anti-IncA and analyzed by microscopy. Deconvolved z-series were used to determine Manders coefficients between PDI and IncA or CERT and IncA. Image analysis was done using ImageJ, macro is provided in supplemental methods. Only single manders coefficient shown above, corresponding to the percentage of CERT that overlaps with IncA (A), or the percentage of IncA that overlaps with PDI (B). Each dot represents a single inclusion, two independent trials were performed with at least 12 inclusions analyzed per condition, per trial. (A) One-way ANOVA with Dunnett’s multiple comparisons test was done comparing different treatment means to the mean of DMSO treated inclusions. The results of the ANOVA were significant with p = 0.0428, however no comparisons were significant using Dunnett’s multiple comparisons test. The coefficients of 4PBA were approaching significance with p = 0.0749, however since the 4PBA sample mean was higher than DMSO this does not suggest that 4PBA is inhibiting membrane contact sites between the ER and inclusion. (B) Results of a one-way ANOVA were not significant (p = 0.0707).
(TIF)

S11 Fig Golgi is disrupted by BFA and FLI-06 but not 4PBA. HeLa cells were infected with *C*. *trachomatis* for 20 hours; 10 μM FLI-06, 5 mM 4PBA, or 3 μg/mL BFA were added from 20–24 hpi, then cells were fixed and processed for immunofluorescence. Golgi marker GM130 (anti-GM130, red) and DNA (DAPI) are shown at 20x magnification. Inclusions marked with stars, scale bar = 32 μm.
(TIF)

S12 Fig Westerns and microscopy for ERES RNAi. (A) Western blots were used to confirm siRNA knockdown at the protein level. HeLa cells were transfected with siRNA oligonucleotides and incubated for 48 hours, then protein levels assessed by western blot. Knockdown was compared to cells transfected with a non-targeting (NT) control oligonucleotide. Beta-actin was used as a loading control. We were unable to detect Sec16A by western blot, so knockdown was confirmed by immunofluorescence microscopy. (B) HeLa cells were transfected with either the Sec16A siRNA oligonucleotide or NT control, incubated for 48 hours, then infected with *C*. *trachomatis* L2. Cells were fixed at 24 hpi and stained with anti-Sec16A, anti-IncA, and DAPI. Images are 20x magnification. In control cells, Sec16A (green in merge) is visible in every cell, while in the Sec16A targeting siRNA treated cells, most cells have no visible Sec16A. Knockdown efficiency was less than 100%, as shown by two cells with visible Sec16A expression (arrows). Scale bars = 30 μm.
(TIF)

S13 Fig Sec12 and Sec31 colocalize after treatment with FLI-06 or 4PBA. HeLa cells were infected with *C*. *trachomatis* and treated with 10 μM FLI-06 or 5 mM 4PBA from 20–24 hpi. Cells were then fixed with ice cold methanol and stained for Sec12 (anti-Sec12, purple in merge), Sec31A (anti-Sec31A, green in merge), or DNA (DAPI, blue in merge). Images are deconvolved merged z-series, scale = 16 μm.
(TIF)

S1 Table Complete proteomic data. (XLSX)

S2 Table IncB-APEX2 proteome statistical analysis and abundance values. (XLSX)

S1 File CellProfiler pipeline used for quantifying ERES association with inclusion supplemental methods. Methods file contains detailed information for siRNA oligonucleotide sequences, qPCR oligonucleotide sequences, ImageJ macros for image quantitation, and R script used for protein network analysis.
(CPPIPE)
