# Supplementary figures and images for: Proximity-dependent proteomics of the Chlamydia trachomatis inclusion membrane reveals functional interactions with endoplasmic reticulum exit sites
Source: PLoS Pathog. 2019 Apr 3;15(4):e1007698. doi: 10.1371/journal.ppat.1007698 (PMC6464245; doi:10.1371/journal.ppat.1007698)

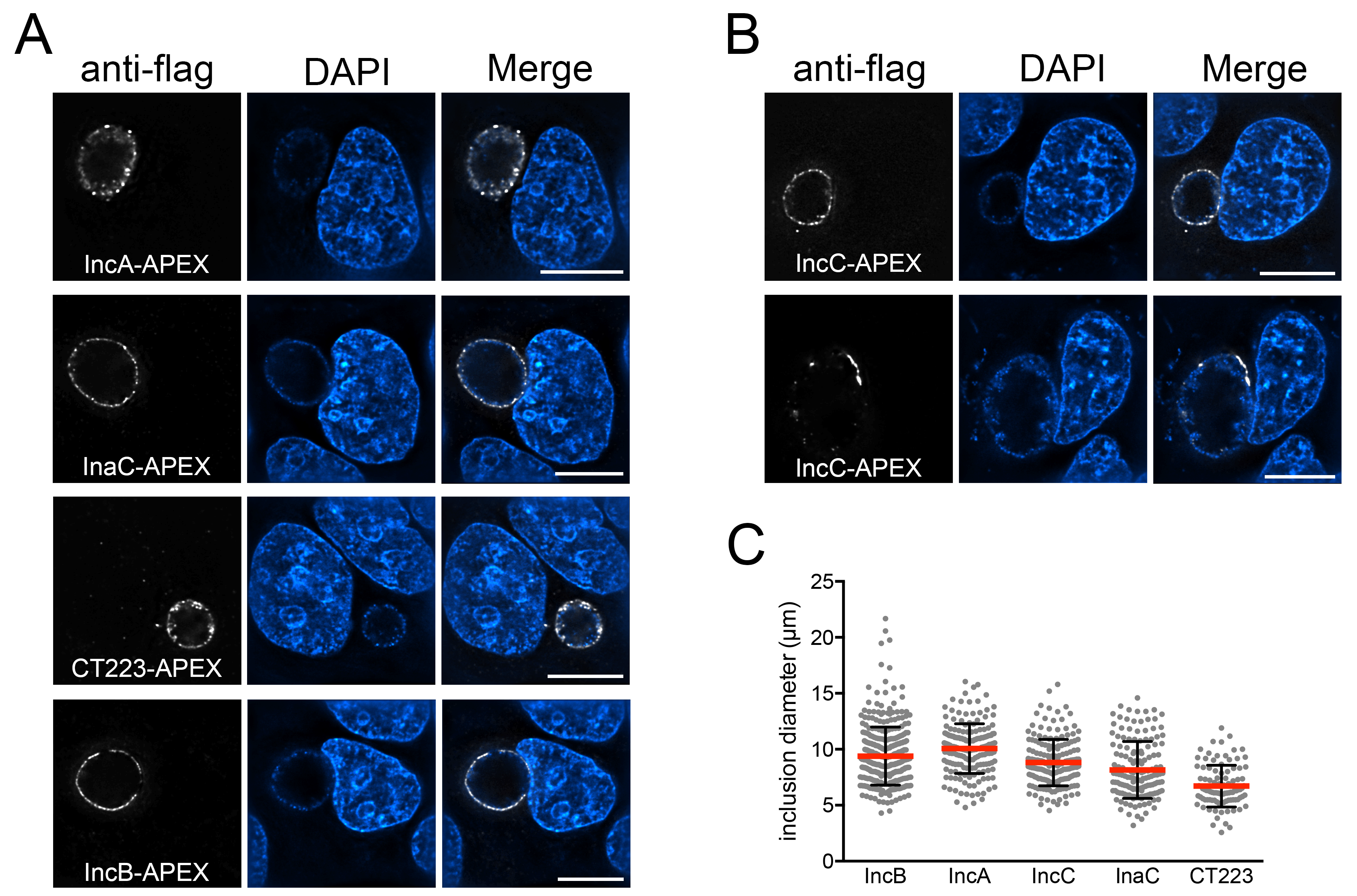

Supplement: S1 Fig — (A) HeLa cells were infected with C. trachomatis transformed with plasmids containing several known Incs fused to flag-APEX2. 1 ng/mL ATc was added at the start of infection, cells were fixed at 24 hpi and stained with anti-flag (white) and DAPI (blue). Images shown are single planes of deconvolved z-series. Scale bars = 16 μm. (B) Cells were infected with C. trachomatis transformed to express IncC-APEX2 with a flag tag, cells were treated as described in A. IncC-APEX2 was sometimes distributed evenly around the inclusion (top images), while in other inclusions it had a microdomain localization pattern (bottom images). Scale bars = 16 μm. (C) Cells were infected and fixed as described in A, and inclusion diameter was measured. Incs listed are referring to the Inc fused to APEX2 in the Chlamydia transformant tested. Each dot represents one inclusion, red line represents mean diameter with SD in black. Mean diameter values in left to right order were 9.40 μm, 10.07 μm, 8.82 μm, 8.16 μm, 6.71 μm. (TIF) [file ppat.1007698.s001.tif]

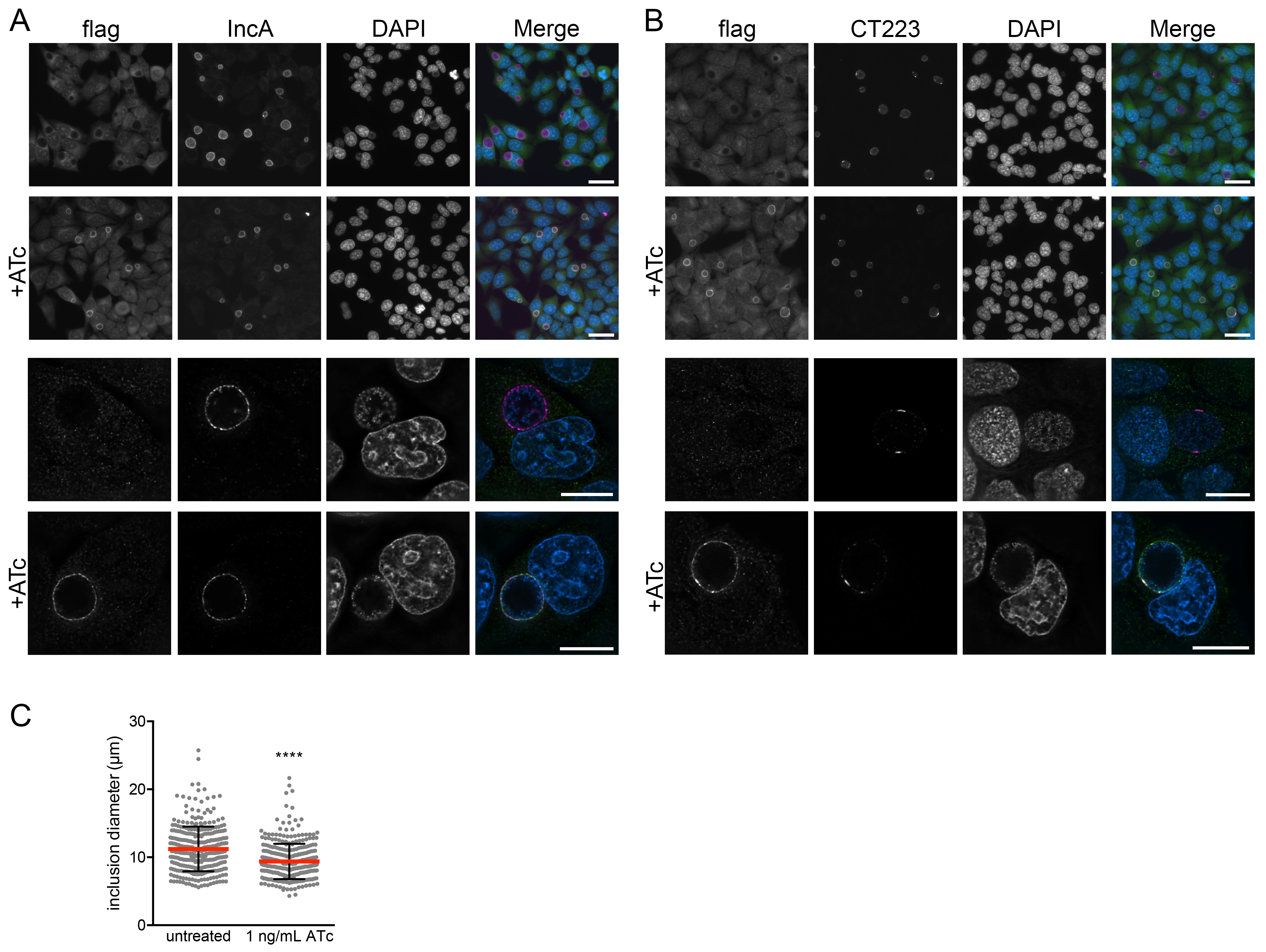

Supplement: S2 Fig — HeLa cells were infected with C. trachomatis transformed with tet inducible, flag tagged, IncB-APEX2 plasmid. Cells were grown in normal conditions or with 1 ng/mL ATc (rows labeled +ATc). At 24 hpi, cells were fixed and stained with anti-flag, anti-IncA (A), or anti-CT223 (B) antibodies and DNA was labeled with DAPI. Images are 20x magnification (Top two rows of A and B, scale bars = 32 μm) or single plane from 60x deconvolved z-series (Bottom two rows of A and B, scale bars = 16 μm). (C) Same samples as described in A, B, plot of inclusion diameters with or without 1ng/mL ATc. Each dot represents one inclusion, measurements taken from two independent experiments. Significance determined by two-tailed Mann-Whitney test, red line shows mean (SD); ****, p < 0.0001. Mean diameter for untreated inclusions was 11.22 μm and 9.40 μm for inclusions treated with 1 ng/mL ATc. (TIF) [file ppat.1007698.s002.tif]

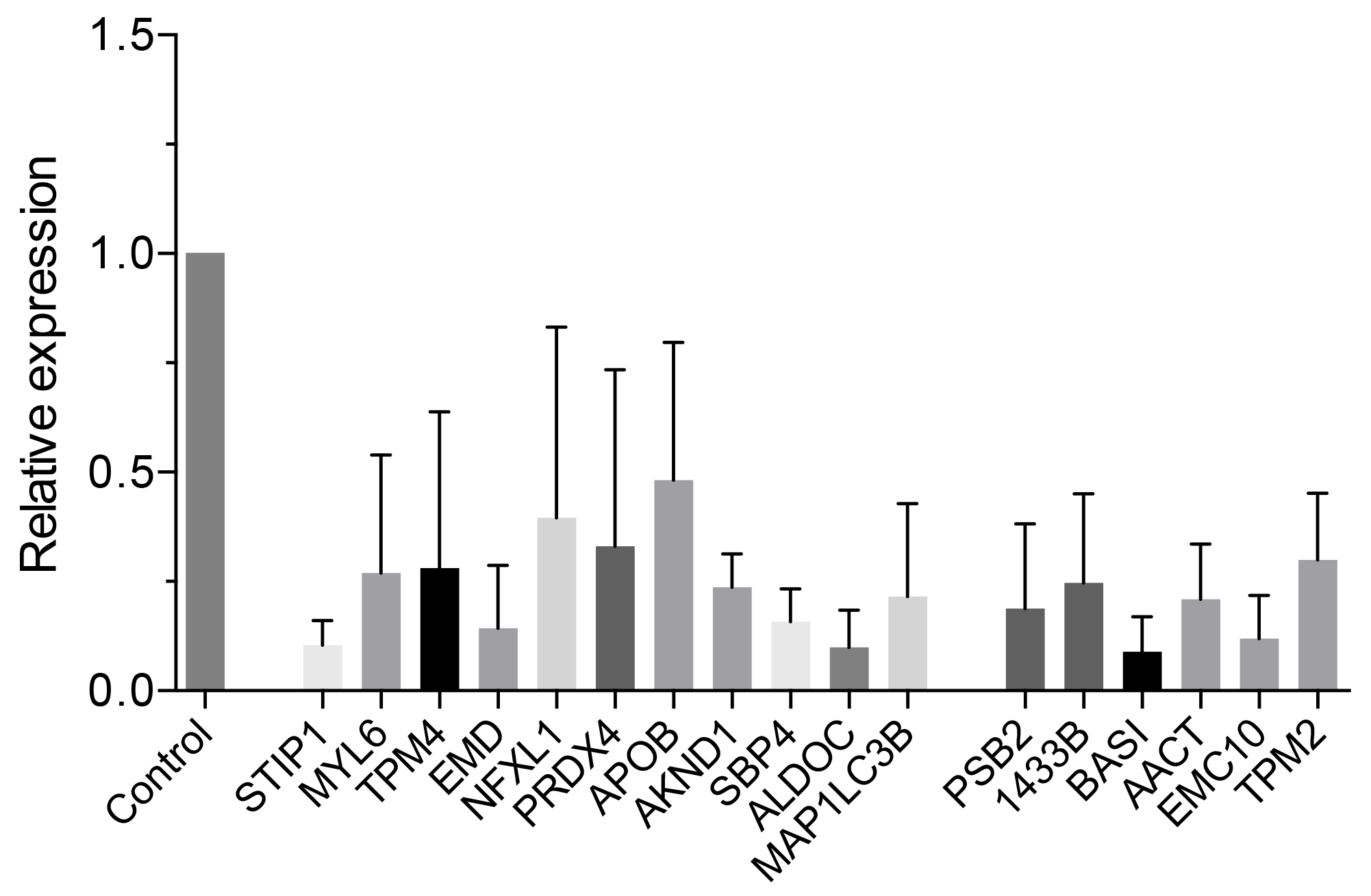

Supplement: S3 Fig — HeLa cells were transfected with siRNA oligos corresponding to the genes listed in graph or non-targeting control. Relative expression was measured using qRT-PCR using the ΔΔCT method at 48 hours post transfection. Order corresponds to effect on IFU, from left to right (not including control) is from highest reduction in IFU to most increased IFU. (TIF) [file ppat.1007698.s003.tif]

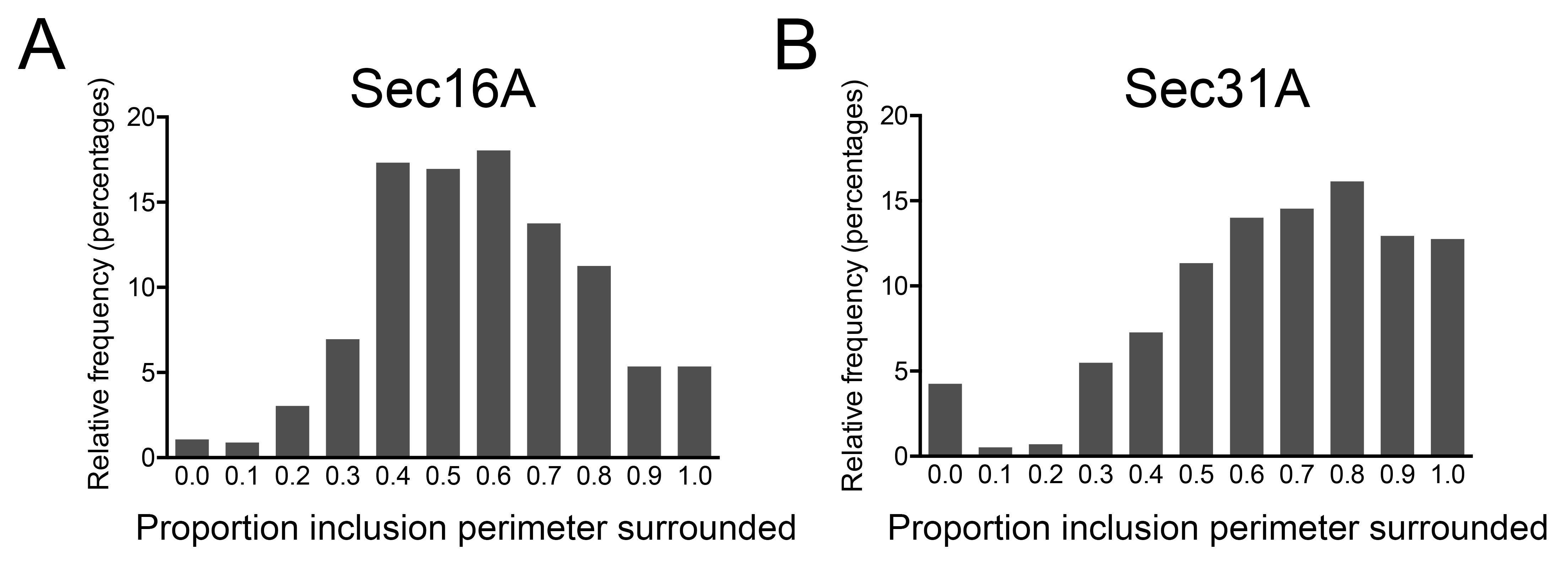

Supplement: S4 Fig — HeLa cells were infected with C. trachomatis L2 and fixed at 24 hpi. Cells were stained with anti-IncA and either anti-Sec16A or anti-Sec31A and 20x images were taken. Concentrated regions of ERES marked by Sec16A (A) or Sec31A (B) were outlined using CellProfiler, complete description of image processing is in supplemental methods [44]. Inclusion perimeters were also outlined using CellProfiler, and the fraction of inclusion perimeter that overlapped the outlined concentrated ERES was calculated. Each inclusion was calculated individually, graph shows data from two independent trials with at least 200 inclusions measured per trial. Percentage refers to percentage of inclusions with specified fraction overlap. (TIF) [file ppat.1007698.s004.tif]

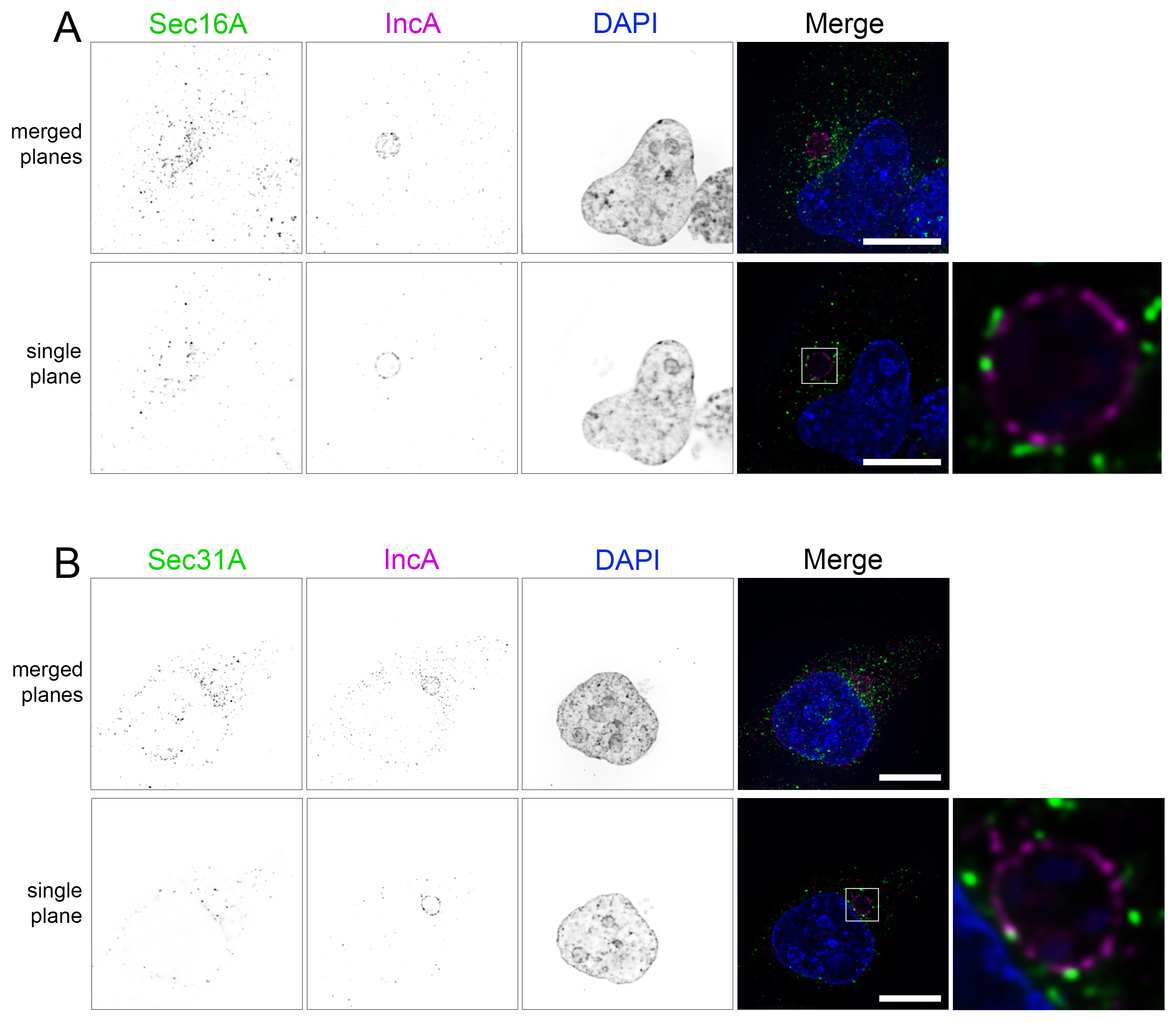

Supplement: S5 Fig — HeLa cells infected with C. trachomatis L2 were fixed at 14 hpi and stained with anti-Sec16A (A) or anti-Sec31A (B), anti-IncA, and DAPI. Top rows of A and B are deconvolved and merged z-series images; bottom rows are single deconvolved planes. Scale bars = 10 μm. (TIF) [file ppat.1007698.s005.tif]

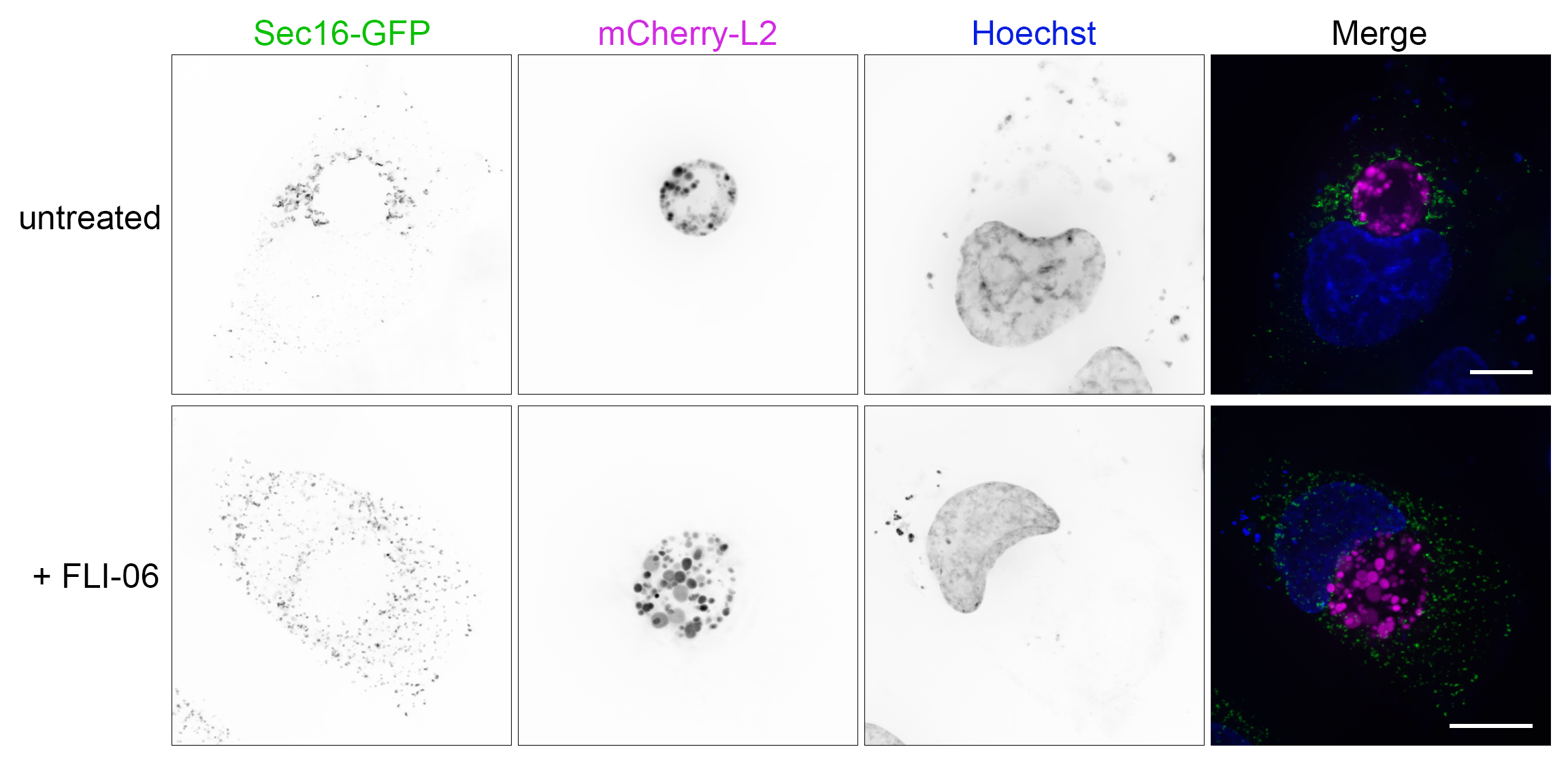

Supplement: S6 Fig — HeLa cells were transfected with a Sec16-GFP plasmid and infected with mCherry expressing C. trachomatis L2. DNA was labeled with Hoechst and cells were imaged live at 24 hpi. In top row, Sec16-GFP shows a similar localization to antibody staining (Fig 5A), with an enrichment near the inclusion membrane. Bottom row shows cells treated with 10 μM FLI-06 from 20–24 hpi, resulting in diffuse Sec16-GFP punctae throughout the cell. Scale bars = 16 μm, images are deconvolved merged z-series. (TIF) [file ppat.1007698.s006.tif]

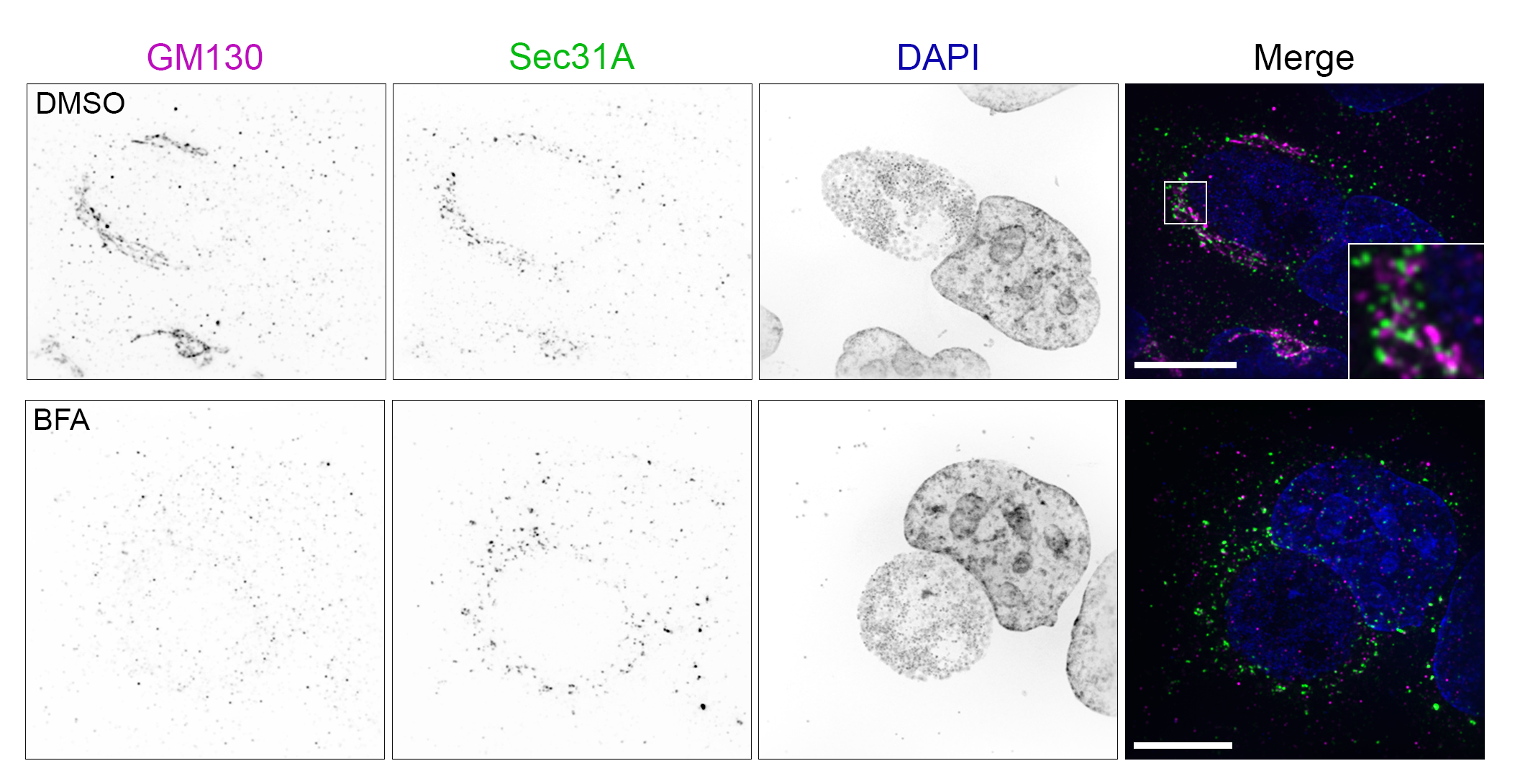

Supplement: S7 Fig — HeLa cells were infected with C. trachomatis and treated with either DMSO or 3 μg/mL BFA from 20–24 hpi. Cells were fixed at 24 hpi and stained with GM130 (anti-GM130, purple in merge) to mark the Golgi, Sec31A (anti-Sec31A, green in merge) to mark ERES, and DAPI for DNA (blue in merge). Scale = 16 μm, images are deconvolved merged z-series. (TIF) [file ppat.1007698.s007.tif]

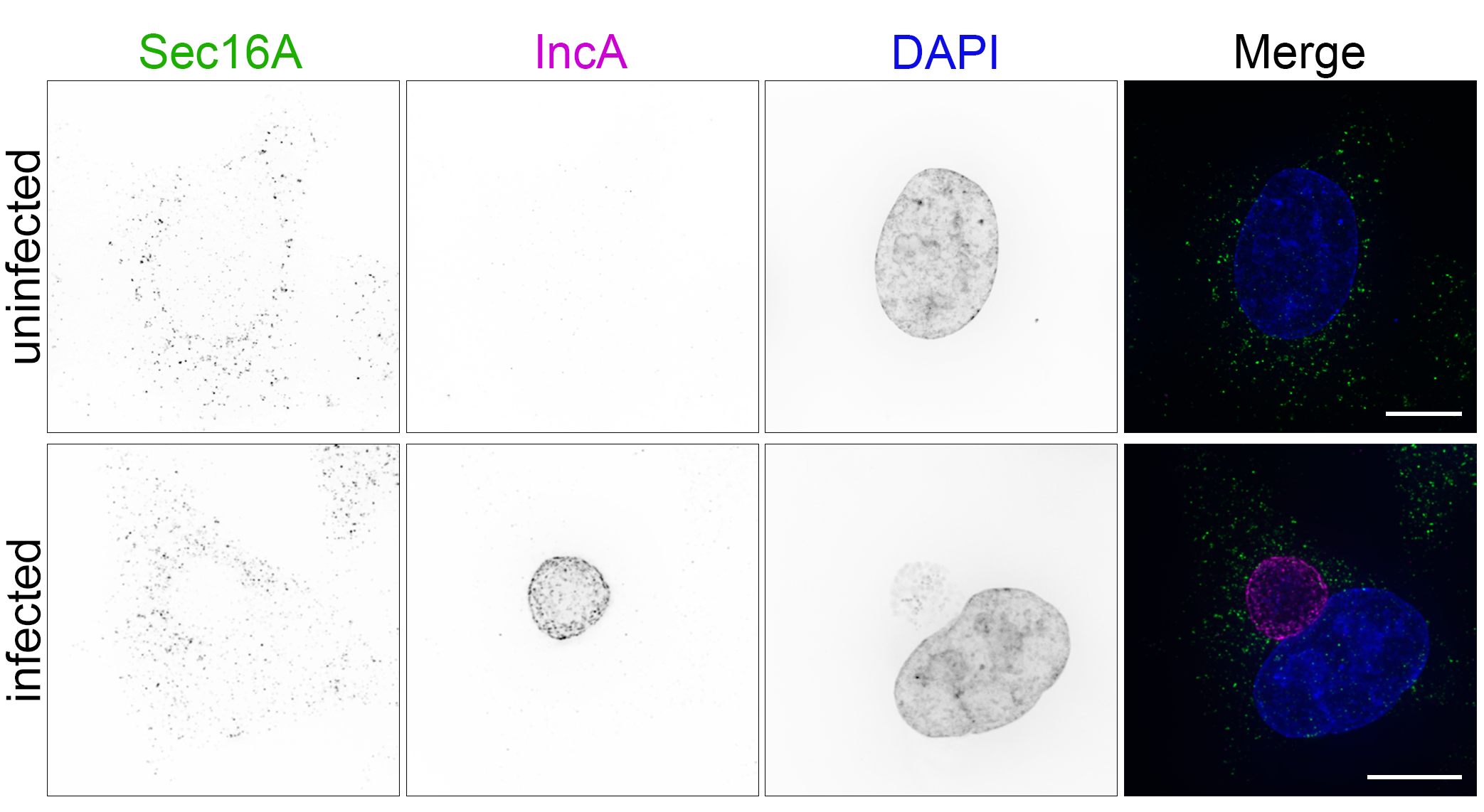

Supplement: S8 Fig — HeLa cells were infected with C. trachomatis and incubated with FLI-06 from 20–24 hpi, then fixed and processed for immunofluorescence. Sec16A (anti-Sec16A, green in merge), IncA (anti-IncA, purple in merge), and DNA (DAPI, blue in merge) were labeled, showing an altered localization of ERES in the presence of FLI-06. Scale = 16 μm, images are deconvolved merged z-series. (TIF) [file ppat.1007698.s008.tif]

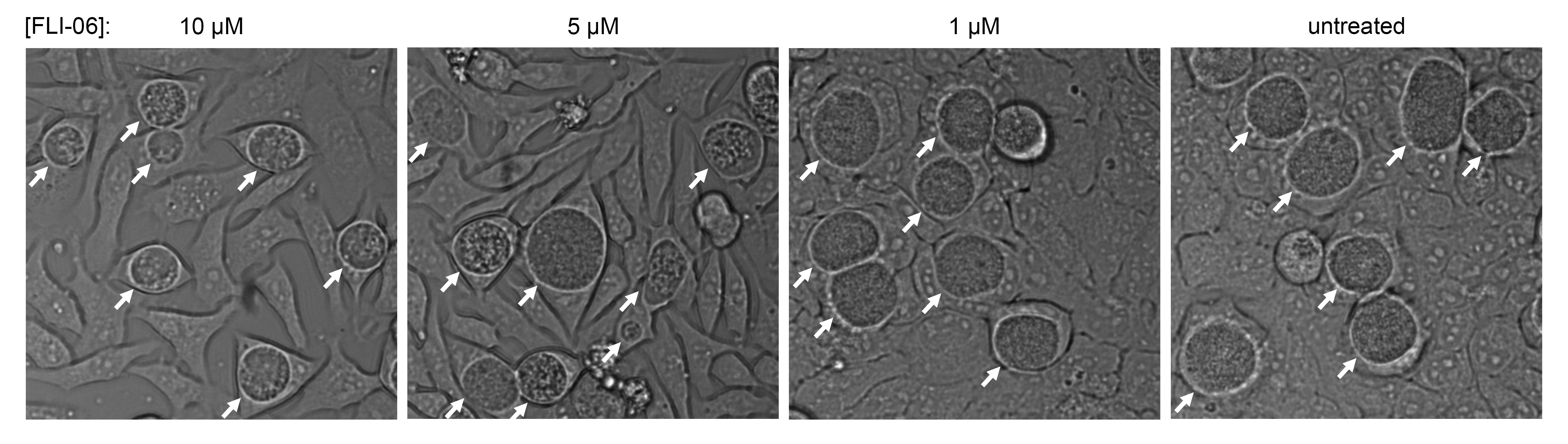

Supplement: S9 Fig — Brightfield images at 20x magnification of infected cells treated with FLI-06 starting at 18 hpi, at indicated concentrations, and measured at 48 hpi. At 10 and 5 μM FLI-06, inclusion morphology appears smaller with bacteria less uniformly distributed, compared to the 1 μM or untreated cells. Arrows indicate inclusions. (TIF) [file ppat.1007698.s009.tif]

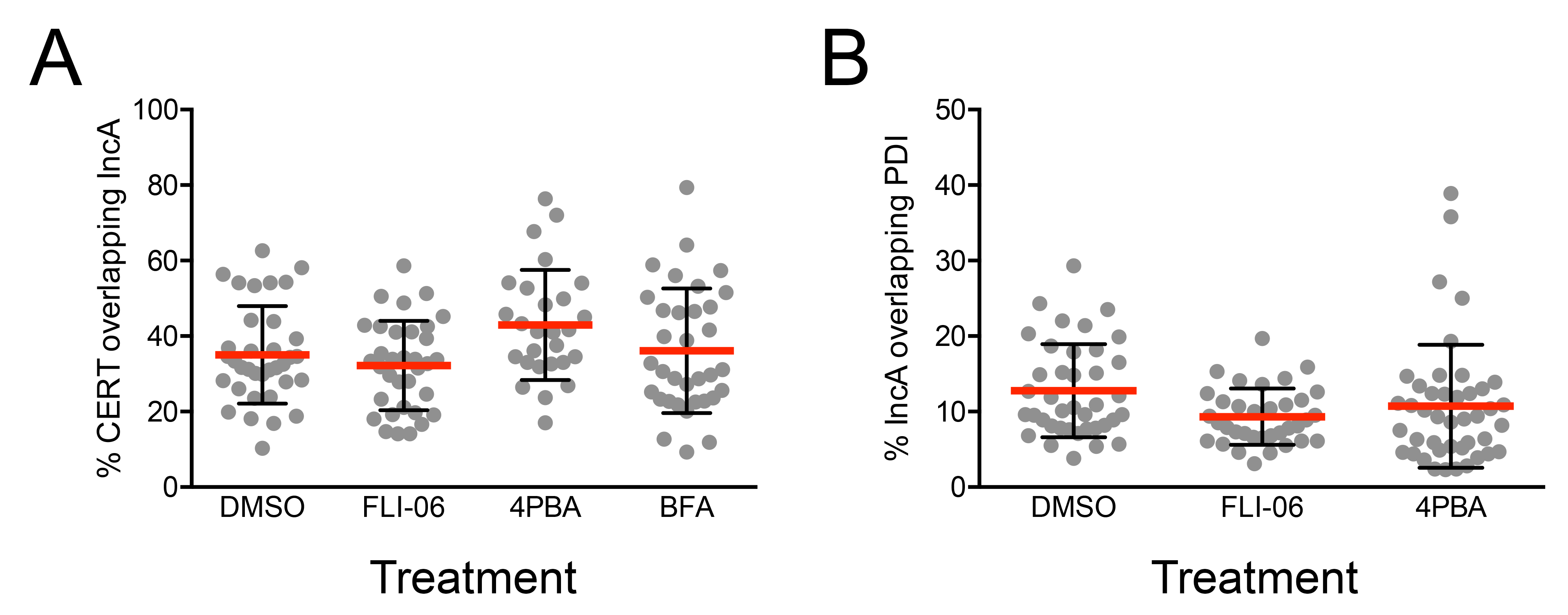

Supplement: S10 Fig — HeLa cells were transfected with plasmids pmScarlet-CERT (A) or pmClover-PDI (B) then infected with C. trachomatis L2 and fixed at 24 hpi. Cells were stained with anti-IncA and analyzed by microscopy. Deconvolved z-series were used to determine Manders coefficients between PDI and IncA or CERT and IncA. Image analysis was done using ImageJ, macro is provided in supplemental methods. Only single manders coefficient shown above, corresponding to the percentage of CERT that overlaps with IncA (A), or the percentage of IncA that overlaps with PDI (B). Each dot represents a single inclusion, two independent trials were performed with at least 12 inclusions analyzed per condition, per trial. (A) One-way ANOVA with Dunnett’s multiple comparisons test was done comparing different treatment means to the mean of DMSO treated inclusions. The results of the ANOVA were significant with p = 0.0428, however no comparisons were significant using Dunnett’s multiple comparisons test. The coefficients of 4PBA were approaching significance with p = 0.0749, however since the 4PBA sample mean was higher than DMSO this does not suggest that 4PBA is inhibiting membrane contact sites between the ER and inclusion. (B) Results of a one-way ANOVA were not significant (p = 0.0707). (TIF) [file ppat.1007698.s010.tif]

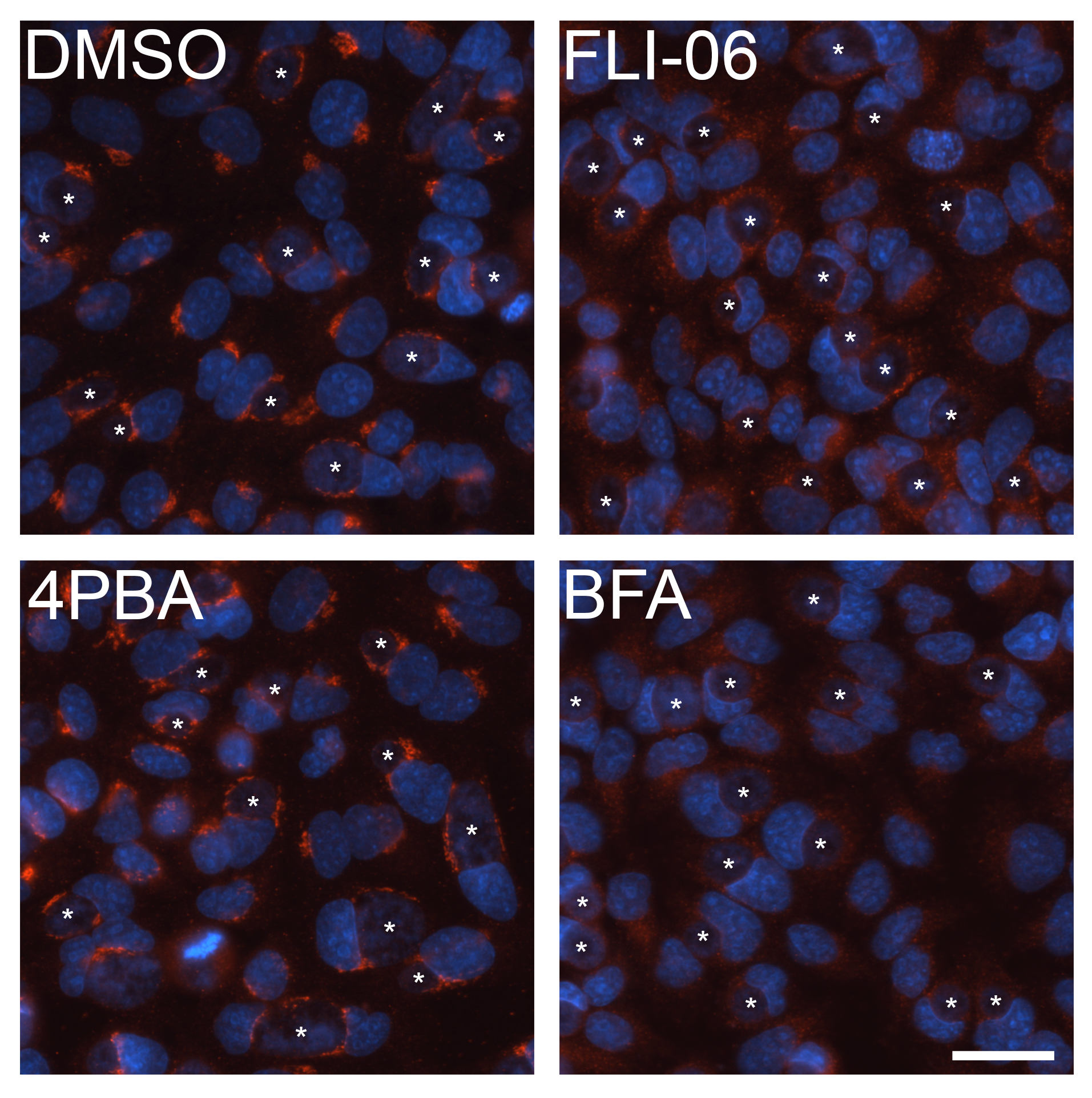

Supplement: S11 Fig — HeLa cells were infected with C. trachomatis for 20 hours; 10 μM FLI-06, 5 mM 4PBA, or 3 μg/mL BFA were added from 20–24 hpi, then cells were fixed and processed for immunofluorescence. Golgi marker GM130 (anti-GM130, red) and DNA (DAPI) are shown at 20x magnification. Inclusions marked with stars, scale bar = 32 μm. (TIF) [file ppat.1007698.s011.tif]

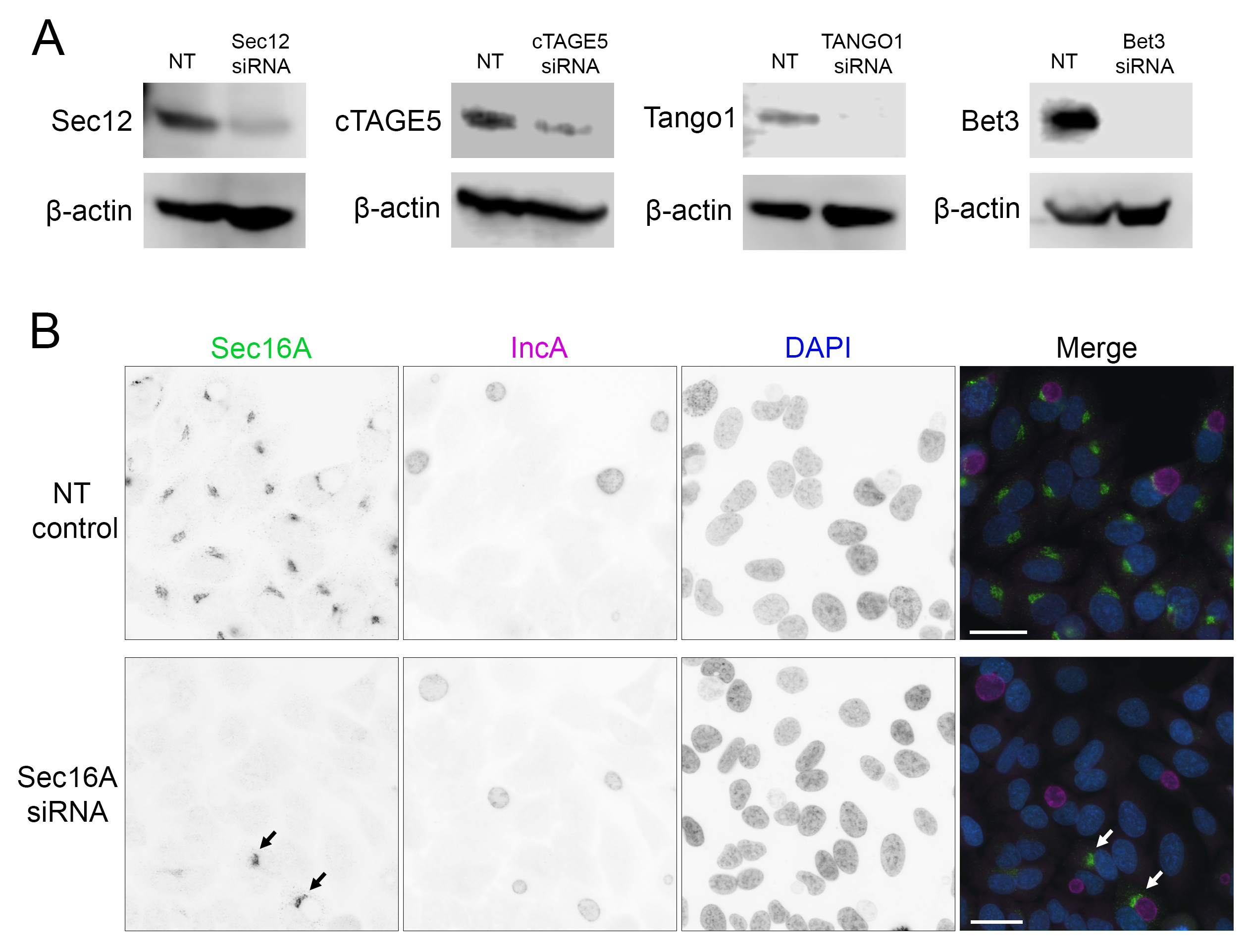

Supplement: S12 Fig — (A) Western blots were used to confirm siRNA knockdown at the protein level. HeLa cells were transfected with siRNA oligonucleotides and incubated for 48 hours, then protein levels assessed by western blot. Knockdown was compared to cells transfected with a non-targeting (NT) control oligonucleotide. Beta-actin was used as a loading control. We were unable to detect Sec16A by western blot, so knockdown was confirmed by immunofluorescence microscopy. (B) HeLa cells were transfected with either the Sec16A siRNA oligonucleotide or NT control, incubated for 48 hours, then infected with C. trachomatis L2. Cells were fixed at 24 hpi and stained with anti-Sec16A, anti-IncA, and DAPI. Images are 20x magnification. In control cells, Sec16A (green in merge) is visible in every cell, while in the Sec16A targeting siRNA treated cells, most cells have no visible Sec16A. Knockdown efficiency was less than 100%, as shown by two cells with visible Sec16A expression (arrows). Scale bars = 30 μm. (TIF) [file ppat.1007698.s012.tif]

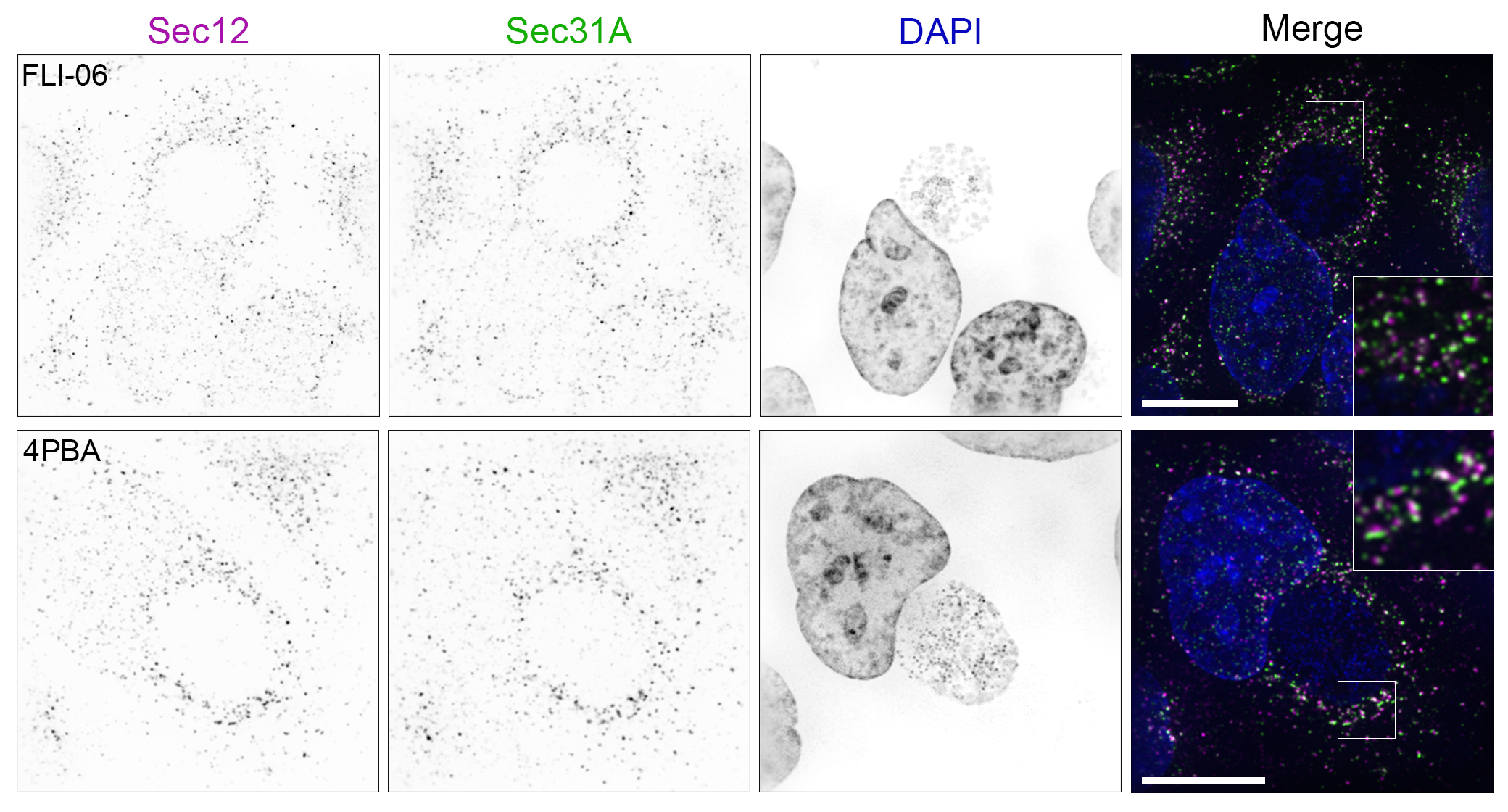

Supplement: S13 Fig — HeLa cells were infected with C. trachomatis and treated with 10 μM FLI-06 or 5 mM 4PBA from 20–24 hpi. Cells were then fixed with ice cold methanol and stained for Sec12 (anti-Sec12, purple in merge), Sec31A (anti-Sec31A, green in merge), or DNA (DAPI, blue in merge). Images are deconvolved merged z-series, scale = 16 μm. (TIF) [file ppat.1007698.s013.tif]
